# The General Transcriptional Repressor Tup1 Is Required for Dimorphism and Virulence in a Fungal Plant Pathogen

**DOI:** 10.1371/journal.ppat.1002235

**Published:** 2011-09-01

**Authors:** Alberto Elías-Villalobos, Alfonso Fernández-Álvarez, José I. Ibeas

**Affiliations:** Centro Andaluz de Biología del Desarrollo, Universidad Pablo de Olavide-Consejo Superior de Investigaciones Científicas, Sevilla, Spain; Virginia Polytechnic Institute and State University, United States of America

## Abstract

A critical step in the life cycle of many fungal pathogens is the transition between yeast-like growth and the formation of filamentous structures, a process known as dimorphism. This morphological shift, typically triggered by multiple environmental signals, is tightly controlled by complex genetic pathways to ensure successful pathogenic development. In animal pathogenic fungi, one of the best known regulators of dimorphism is the general transcriptional repressor, Tup1. However, the role of Tup1 in fungal dimorphism is completely unknown in plant pathogens. Here we show that Tup1 plays a key role in orchestrating the yeast to hypha transition in the maize pathogen *Ustilago maydis.* Deletion of the *tup1* gene causes a drastic reduction in the mating and filamentation capacity of the fungus, in turn leading to a reduced virulence phenotype. In *U. maydi*s, these processes are controlled by the *a* and *b* mating-type loci, whose expression depends on the Prf1 transcription factor. Interestingly, Δ*tup1* strains show a critical reduction in the expression of *prf1* and that of Prf1 target genes at both loci. Moreover, we observed that Tup1 appears to regulate Prf1 activity by controlling the expression of the *prf1* transcriptional activators, *rop1* and *hap2*. Additionally, we describe a putative novel *prf1* repressor, named Pac2, which seems to be an important target of Tup1 in the control of dimorphism and virulence. Furthermore, we show that Tup1 is required for full pathogenic development since *tup1* deletion mutants are unable to complete the sexual cycle. Our findings establish Tup1 as a key factor coordinating dimorphism in the phytopathogen *U. maydis* and support a conserved role for Tup1 in the control of hypha-specific genes among animal and plant fungal pathogens.

## Author Summary

Fungal plant pathogens cause serious damage to crops with huge social and economic consequences. To cause disease, many such fungi need to change their morphology between a yeast-like, unicellular form and a filamentous state. This change, known as dimorphism, is tightly controlled by complex genetic pathways to ensure successful pathogenic development. In animal pathogens, one of the most important genes controlling dimorphism is Tup1. In plant pathogens, however, the role for this gene is completely unknown. In this work, we describe the role of Tup1 in the dimorphism and virulence of *Ustilago maydis*, the plant fungal pathogen that causes maize smut disease. We show that mutant *U. maydis* cells lacking Tup1 are unable to properly change between yeast-like and filamentous forms, thus compromising its virulence. We look at the underlying genetic pathways, and find that Tup1 regulates key genes known to regulate dimorphism. We also show that Tup1 is essential for the production of mature fungal spores, which normally allow the fungus to disperse and infect new plants. Our results show that Tup1 is a key element in the control of both infectious and dispersible fungal forms and supports an evolutionary-conserved role for this gene in the regulation of dimorphism among animal and plant pathogenic fungi.

## Introduction

Dimorphism, the capacity of certain fungi to change their morphology between yeast-like growth and a filamentous state in response to environmental signals, is frequently associated with the virulence of both animal and plant pathogenic fungi [1]–[6]. This morphological conversion is controlled by several conserved signaling pathways, such as the cyclic AMP-protein kinase A pathway and a mitogen-activated protein (MAP) kinase cascade [4], [6]–[10]. Another well known transcriptional regulator controlling dimorphism is the general transcriptional repressor Tup1, which is conserved from fungi to mammals [11]–[16]. The mechanism of action for Tup1 has been best studied in the yeast *Saccharomyces cerevisiae*. In this fungus, Tup1p forms a transcriptional co-repressor complex with Ssn6p, a protein that contains tetratricopeptide repeat (TPR) motifs known to mediate protein-protein interactions [17]–[20]. Neither Tup1p nor Ssn6p have direct DNA binding activity and their role in transcription depends on their recruitment to promoters by specific DNA binding proteins [18], [21]. Tup1p repression mechanisms include the interaction with RNA polymerase II holoenzyme components and the alteration of chromatin structure through interaction with histones H3 and H4 and histone deacetylases [22]–[26]. Tup1p controls *S. cerevisiae* dimorphism in both haploid and diploid strains. Deletions of *TUP1* result in reduced haploid invasive growth and reduced diploid pseudohyphal growth, which are considered the filamentous forms of this yeast [11].

Although the role of Tup1 in fungal dimorphism seems conserved, the way it controls this process frequently differs between fungi. The deletion of *tup1* from the animal pathogens *Candida albicans*, *Penicillium marneffei* and *Cryptococcus neoformans* give clear examples of this variability. In *C. albicans*, the homozygous mutant for *TUP1* shows a constitutive filamentation phenotype, in contrast to the situation described for *S. cerevisiae*, and reduced virulence [11]. In *P. marneffei*, however, *tupA* is required for the maintenance of its filamentous form, negatively regulating yeast morphogenesis instead of filament formation [12]. In the case of *C. neoformans*, *TUP1* is required for the formation of dikaryotic hyphae due to a mating defect of *TUP1* mutant strains, and for virulence [27], [28]. In addition, the molecular mechanisms and genetic pathways by which Tup1 acts in fungal dimorphism are poorly understood in most species [7], [12], [27]–[33]. This role of Tup1 in regulating the dimorphic transition is completely unknown in plant pathogenic fungi, which require different morphogenetic changes to successfully colonize their hosts and cause disease. The only data that might link Tup1 to a role in plant fungal dimorphism are a study into the role of *sql1*, a gene functionally homologous to *S. cerevisiae SSN6*, in *U. maydis*. Here overexpression of truncated forms of Sql1 was shown to induce morphological changes in this fungus [34].

The corn smut fungus *Ustilago maydis* is a well established model for studying dimorphism and virulence in plant pathogens [35]–[38]. Pathogenic development of this fungus initiates with the transition from yeast-like growth to the formation of polar filaments on the plant leaf surface. Control of this process relies on a tetrapolar mating system consisting of the biallelic *a* and the multiallelic *b* loci. Only strains differing in the allelic composition at both loci can successfully form and maintain the infectious filamentous form of the fungus [39]. Locus *a* encodes the pheromone-receptor system that allow cells from different mating types to detect each other, form conjugation tubes, and fuse [40], [41]. Locus *b* is then responsible for determining the fate of the resulting dikaryon. This locus encodes a pair of homeodomain transcription factors, bE and bW, that form a compatible heterodimer if proceeding from different alleles, triggering filamentation and pathogenicity [42], [43]. Upon dikaryon filament formation, the hypha tip differentiates to form a specialized structure for plant penetration, known as the appressorium [44], [45]. Once inside the plant, mycelium expansion takes place, leading to the formation of plant tumors. In these tumors, fungal nuclei fuse prior to the separation and rounding up of each hyphal section to form diploid spores. In favorable conditions spores germinate in a meiotic process that forms new haploid cells [46].

**Figure 1:**
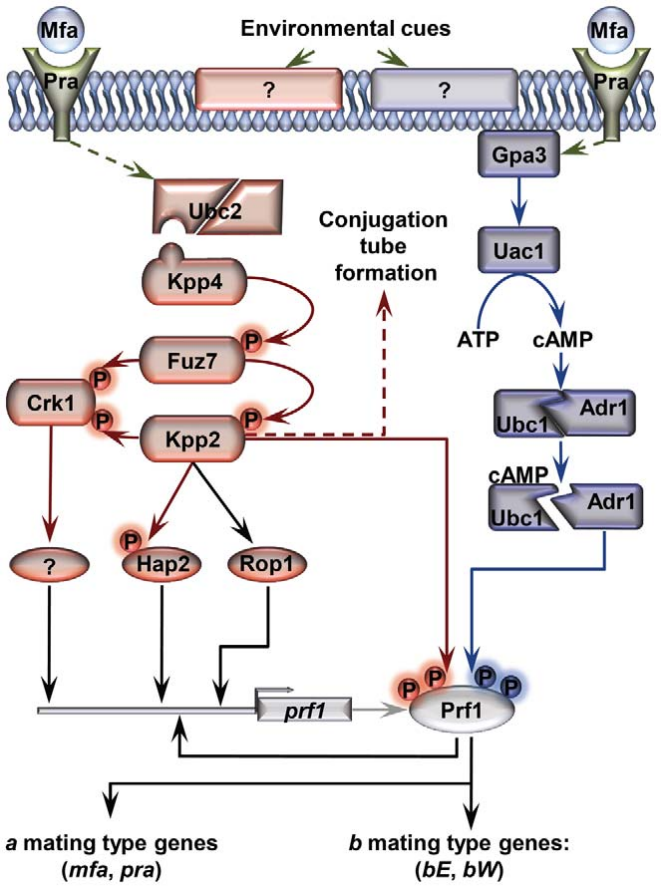
Schematic representation of the regulation of *U. maydis* mating-type gene expression. Pheromone (Mfa) recognition by the receptor (Pra) of the opposite mating type, together with environmental cues sensed by unknown receptors (represented by question marks), result in the activation of the cAMP (blue) and MAP kinase (red) pathways. The central core of the MAP kinase module is composed of Kpp4 (MAPKKK), Fuz7 (MAPKK) and Kpp2 (MAPK), and the alternative MAP kinase, Crk1. Once both pathways have been induced, the downstream transcription factor Prf1 becomes transcriptionally and post-translationally activated and the expression of *a* and *b* mating-type genes takes place. Transcriptional control of *prf1* depends on Rop1, Hap2, a putative unknown factor induced by Crk1, and Prf1 itself. Activation of the MAP kinase module in compatible haploid FB1 or FB2 strains also leads to the formation of conjugation tubes through a Prf1 independent pathway (discontinuous red arrow). Transcriptional regulation is indicated by black arrows. Scheme adapted from [38].

The highly conserved cAMP and MAP kinase pathways play a central role in the control of several of the morphological changes required during *U. maydis* pathogenic development [47]–[51]. Both of these pathways are activated following the recognition of pheromones by receptors of opposite mating types during the yeast to infective hyphae transition, resulting in the transcriptional and post-translational activation of the Prf1 transcription factor [47], [51]–[53]. Once activated, Prf1 promotes the expression of *a* and *b* loci genes (for review see [38]) (Figure 1). Thus, *U. maydis* integrates the inputs that activate both pathways through Prf1 to promote the *b*-dependent infectious form of the fungus. In the animal pathogen *C. albicans,* cAMP and MAP kinase pathways induce filamentous growth by promoting the activation of Efg1 and Cph1 transcriptional regulators, respectively, that extend down to hypha-specific target genes [2], [7], [54]–[56]. Control of filamentation in this fungus also requires the transcriptional repression of hypha-specific genes via Tup1, which acts through a third parallel pathway involving Rfg1 and Nrg1 transcriptional regulators [7], [29]–[33]. In *U. maydis*, as a plant pathogenic fungus, it is unknown whether or not Tup1 plays a role in dimorphism and virulence. Analyzing the function of Tup1 in this plant pathogen could help better understand how it acts within the genetic pathways controlling these processes in different biological contexts.

In this work, we explore the roles of Tup1 during the life cycle of the maize pathogen *U. maydis*. We demonstrate that *tup1* is required for normal mating and filament formation in this fungus and that it controls these processes by transcriptional activation of the Prf1 transcription factor through at least two of its direct regulators. Additionally, we show that *tup1* is essential for full pathogenic development, affecting tumor formation and spore production. Our results indicate that Tup1 represents a key factor for the regulation of the pathogenic filamentous and dispersible spore forms of the corn smut fungus *U. maydis*.

## Results

### Identification of the *U. maydis tup1* homologue

**Figure 2:**
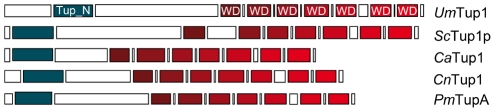
Comparison of conserved protein domains between different members of the Tup1 family of transcriptional repressors. Conserved structure of Tup1 proteins in *U. maydis* (*Um*Tup1), *S. cerevisiae* (*Sc*Tup1p), *C. albicans* (*Ca*Tup1), *C. neoformans* (*Cn*Tup1) and *P. marneffei* (*Pm*TupA) (for accession numbers see Methods). Domains according to InterPro (Pfam) and functionally characterized in *S. cerevisiae* [19], [57] are shown. All the domains described for *Sc*Tup1 are conserved in the *U. maydis* Tup1 protein, including the N-terminal Tup_N domain, required for Ssn6p binding (blue square), seven WD40 domains in the C-terminal region (red tone squares), and a less conserved central region.

To identify Tup1 homologues in *U. maydis* we performed a blast search against the MIPS *U. maydis* database (MUMDB) proteome using Tup1p from the *S. cerevisiae* database (SGD) as the query sequence. A *U. maydis* protein sequence, um03280, with an e-value of 9.5e-81 and 66% similarity to *S. cerevisiae* Tup1p, was retrieved. This sequence, already annotated in MUMDB as Tup1, shows homology to Tup1 proteins from other fungi; including the animal pathogens *C. albicans* (67% similarity), *C. neoformans* (73%) and *P. marneffei* (75%) (all data in Table S1). A sequence alignment of Tup1 proteins from these organisms revealed a number of conserved domains, based on *S. cerevisiae*: (1) the tup_N domain, located in the N-terminal region, which is known to be required for Tup1p/Ssn6p complex formation; (2) seven WD40 domain repeats in the C-terminal region, that mediate protein-protein interactions and (3) a poorly conserved central region, which possesses histone binding activity in *S. cerevisiae* [24], [57], [58] (Figure 2, Table S2 and Figure S1).

### Tup1 is required for full pathogenic development

To test if Tup1 has a role during the *U. maydis* life cycle, we generated deletion mutants for *tup1* in both mating compatible strains, FB1 and FB2, replacing the *tup1* open reading frame with the carboxin resistance cassette from pMF1-c [35]. Examination of cell growth and morphology did not reveal any statistically significant differences in either of the *tup1* mutants (Figure S2).

**Figure 3:**
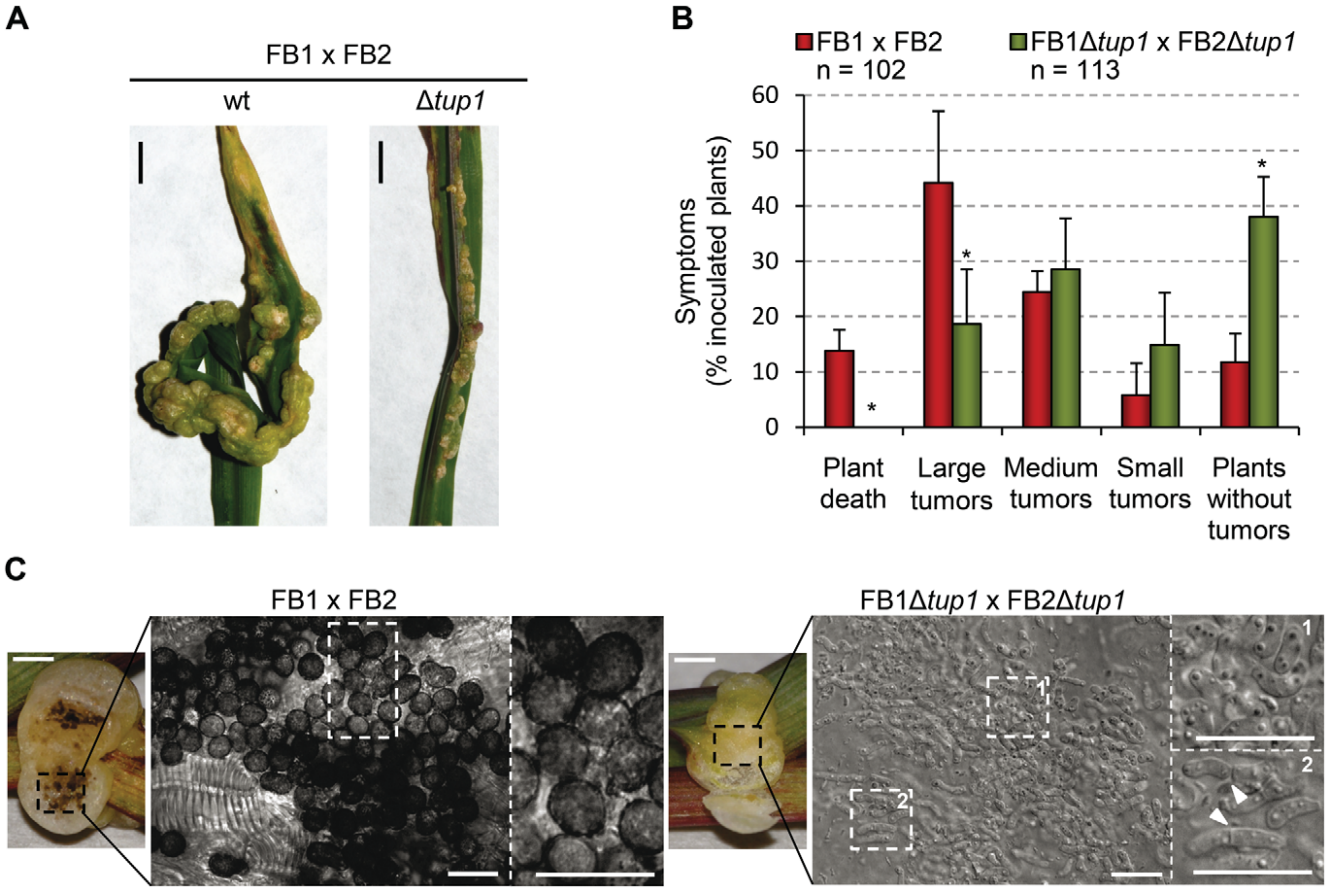
*tup1* is required for full pathogenic development. (A) Representative images showing the most prevalent tumor category for wild-type and *tup1* mutant infected plants. (B) Disease symptoms caused by wild-type and *tup1* mutant strains are shown. Strains are indicated within the color legend. The total number of infected plants (n) is indicated below each strain combination. Symptoms were scored 14 days post-inoculation. Categories correspond to: large tumors (>5 mm), medium tumors (1–5 mm), small tumors (<1 mm). Mean values of three independent experiments and the standard deviation are shown. Asterisk (*) represents statistically significant differences in regard to the wild-type strain. (C) *tup1* mutant spore development phenotypes 21 days post-infection. Left: picture of similarly sized tumors developed by the indicated strains. Strong spore formation is evident by dark coloration inside the tumor. Right: tumor sample analyzed by optical microscopy. Spores were present in tumors induced by wild-type strains. Hyphae at fragmentation or rounded cell formation stages were seen (arrowheads) in the *tup1* mutant-induced tumors. Mature spores were not observed (scale bar  = 20 µm).

Since the *U. maydis* life cycle is intrinsically linked to its host, we assayed the virulence of *tup1* deletion strains. For this purpose, we infected seven day old maize seedlings with compatible mixtures of either wild-type or Δ*tup1* fungi, and scored tumor formation 14 and 21 days post-infection (dpi). We noticed a considerable reduction in the number of Δ*tup1* infected plants that developed tumors compared to wild-type infections. Moreover, the size of tumors developed by Δ*tup1* strains were also considerably reduced (Figure 3A, 3B, and Figure S3). In addition, we observed reduced plant mortality for *tup1* mutant infections, with no dead plants observed at 14 dpi and only 11% mortality versus 57% for the wild-type strain 21 dpi. (Figure 3B and Figure S3).

To ascertain whether *tup1* mutants are able to complete the sexual cycle we assayed infected plants for the presence of spores 21 dpi. Interestingly, while we found large numbers of spores in wild-type tumors, we could not find spores in *tup1* mutant infected plants. Microscopy analysis of the Δ*tup1* induced tumors revealed that none of the fungal hyphae observed had progressed beyond the rounded cell formation stage that occurs just before spore maturation [46] (Figure 3C).

These results indicate that *tup1* is required for full pathogenic development of *U. maydis* and support a conserved role for *tup1* in the virulence of animal and plant fungal pathogens.

### Δ*tup1* cells are impaired in mating and infective filament formation

**Figure 4:**
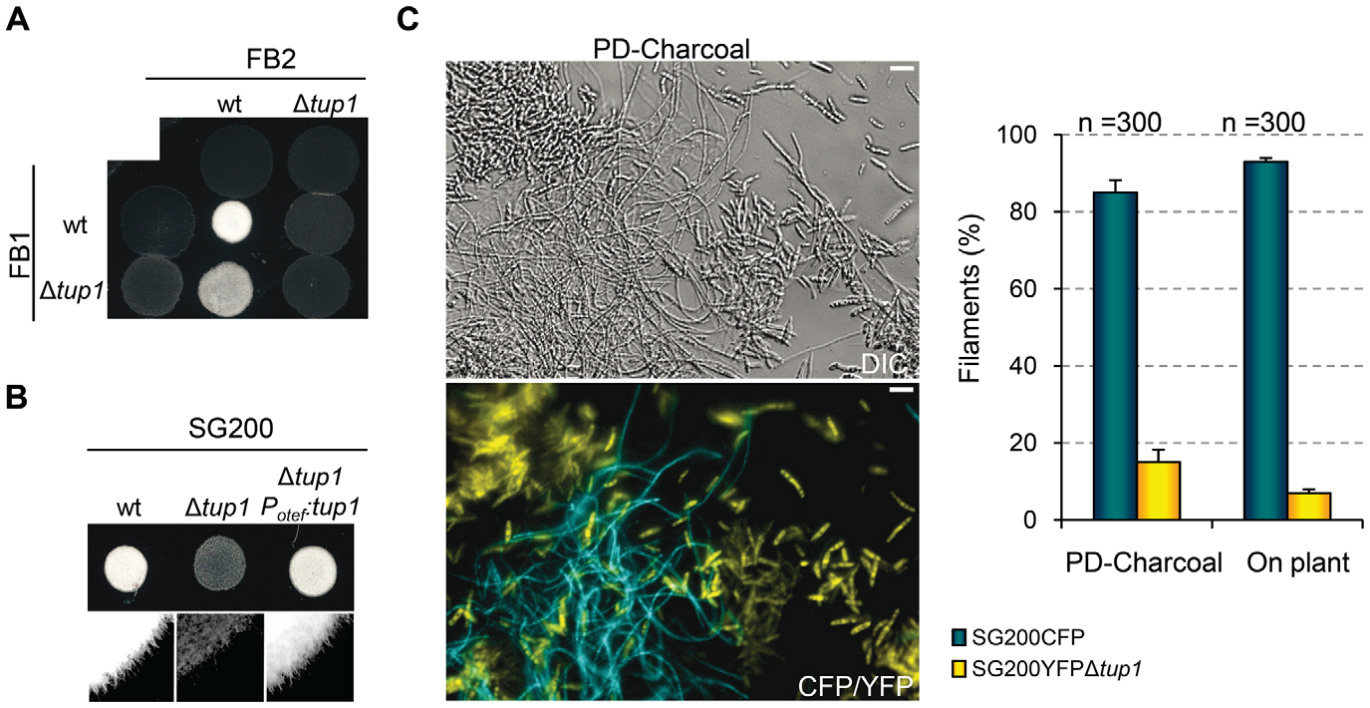
*tup1* is required for mating. (A) Mating between compatible *U. maydis* strains. The strains indicated (top/left) were spotted either alone or in combination and incubated on PD-charcoal plates for 24 hours at 25°C. A white fuzzy colony appearance is indicative of successful mating and the formation of aerial dikaryotic hyphae. (B) Filament formation in SG200 and SG200Δ*tup1* strains. The indicated strains were spotted alone on PD-charcoal plates. The presence of white fuzzy colonies indicates the formation of filaments. (C) Quantification of filamentation defects in the *tup1* deletion strain. A mixture with equal number of cells from SG200CFP and SG200YFPΔ*tup1* were spotted onto charcoal plates or inoculated into maize plants. Image on the left represents the filamentation capacity of both strains on charcoal containing media. Scale bar represents 20 µm. The chart on the right indicates the number of filaments that corresponded to each strain in charcoal plates or on the plant leaf surface. Strains are indicated within the color legend. The total number of filaments counted (n) is indicated above each pair of columns. Mean values of three independent experiments and the standard deviation are shown.

During *U. maydis* plant infection, multiple morphological changes of the fungus are required (for review see [38]). To ascertain which steps of the infectious process are responsible for the decreased amount and size of tumors generated by *tup1* mutants, we first determined the extent to which they were able to successfully undergo mating and develop dikaryon filaments. To test this, we co-spotted compatible combinations of *tup1* mutants and wild-type strains on PD-Charcoal plates, where the appearance of “fuzzy” white colonies indicates successful mating and the formation of dikaryon filaments. As shown in Figure 4A, crosses between *tup1* mutants were unable to form white fuzzy colonies, indicating a recognition or fusion defect between compatible partners, or a post-fusion filamentation defect. Similarly, crosses between *tup1* mutants and compatible wild-type strains also showed fuzzy colony formation defects. Filamentation was partially affected when FB1Δ*tup1* was crossed with wild-type FB2, showing an intermediate phenotype between wild-type and Δ*tup1* crosses. In contrast, the FB1 and FB2Δ*tup1* cross showed the same loss of fuzzy colony phenotype as the double mutant cross. In order to check whether the differences observed in FB1Δ*tup1* and FB2Δ*tup1* strains could lead to different rates of tumor formation, we performed a plant infection assay using FB1 vs FB2Δ*tup1* and FB1Δ*tup1* vs FB2 crosses. As shown in Figure S4A the infection rates of these two strains were similar and slightly different to the rates observed for the cross of both wild type strains.

In addition, we analyzed white fuzzy colony formation in a SG200 background, which is able to form the infective hypha without the necessity of mating with a compatible partner, because of the presence of an active bE1/bW2 heterodimer and a constitutively expressed *mfa2* gene [59]. Significantly, SG200Δ*tup1* did not generate fuzzy colonies on charcoal plates, suggesting a post-fusion role for *tup1* (Figure 4B). In order to quantify the phenotype, we performed a filamentation assay by co-spotting SG200CFP [60] and SG200YFPΔ*tup1* labeled strains on PD-charcoal plates. After fuzzy colony formation, colony samples were used for the quantification of filaments formed by each strain. As shown in Figure 4C, 80% of the filaments corresponded to the wild-type strain, while only 20% belonged to the mutant. Maize infection experiments with *tup1* mutants in the SG200 background revealed similar virulence defects to what we had observed in FB1 and FB2 backgrounds (Figure S5A and S5B). Insertion of a single copy of *tup1* under the control of the constitutive *otef* promoter in the *ip* locus [34] of SG200Δ*tup1*, restored its filamentation and pathogenic capacity, indicating successful complementation (Figure 4B, Figure S5A and S5B). Moreover in the case of the FBD11 diploid strain, which also do not need to mate with a compatible partner to cause virulence, the heterozygous mutant FBD11Δ*tup1*/*tup1* and the homozygous FBD11Δ*tup1*/Δ*tup1* were almost completely avirulent in leaf infection experiments (Figure S4B and S4C). Because of the reduced infection capacity of the FBD11 wild-type strain, we also performed flower infections (where we usually observe bigger tumors) with these strains to better reflect the differences between them. This experiment revealed big tumors in the wild-type strain, medium tumors in the heterozygous and small tumors in the homozygous mutant strains (Figure S4D and S4E).

These results point to a post-fusion filamentation defect as a plausible reason for the impaired pathogenicity of Δ*tup1* strains. However, it has been reported that mating or filamentation defects on PD-Charcoal plates are not always conserved on the plant leaf surface [61]. To check this, we co-infected 7 day old maize seedlings with the labeled strains, SG200CFP and SG200YFPΔ*tup1* and quantified filament formation on the leaf surface. As shown in Figure 4C (on plant columns), the filamentation defect seen on charcoal containing media was also apparent on the leaf surface, with only around 5% of the filaments formed corresponding to the mutant strain.

**Figure 5:**
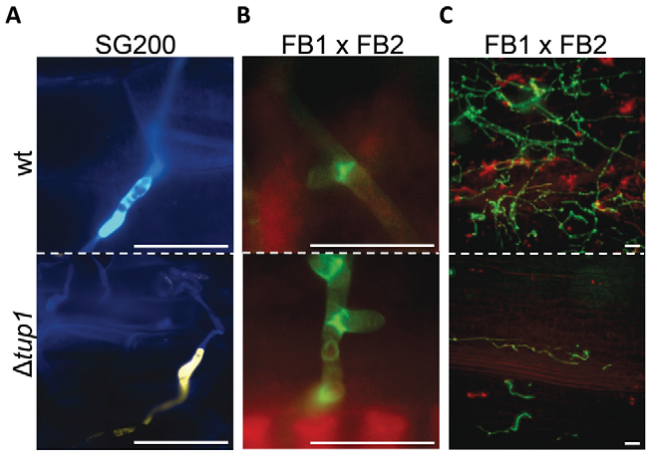
Appressorium, clamp-like cells formation and mycelium expansion of *tup1* mutants. (A) Appressorium formed by wild-type SG200CFP and SG200YFPΔ*tup1* strains. (B) Clamp-like cells formed by wild-type and *tup1* mutant cells 2 dpi. (C) Visualization of mycelium expansion inside the plant tissue of the indicated strains 2 dpi. Infected leaf samples were stained with WGA-AF and propidium iodide (see Methods). Scale bars represent 20 µm.

Finally, to check whether *tup1* could also be implicated in other morphological changes required during the *U. maydis* infection process, we checked for appressoria formation and the presence of clamp-like cells during mycelium expansion in *tup1* mutant strains. We observed that both of these structures were formed in the deletion mutants for *tup1* (Figure 5A and 5B), although at lower frequency than the wild type, which is very likely a consequence of the filament formation defect showed by these mutants. The frequency of appressoria formation by SG200YFPΔ*tup1* was reduced to a similar extent as filament formation (Figure S5C), and mycelium expansion was reduced in Δ*tup1* infected plants at 2 dpi (Figure 5C). These results, together with the capacity, albeit reduced, of *tup1* mutants to induce tumors in maize, suggest that those *tup1* mutant cells that overcome the filamentation defect are then able to undergo the morphological changes required for plant penetration and expansion. Thus, the role of *tup1* in the morphological changes that occur during *U. maydis* infection seems to be specific to the yeast-to-hypha transition.

### Induction of the *b* locus restores the filamentation defect of *tup1* mutants

**Figure 6:**
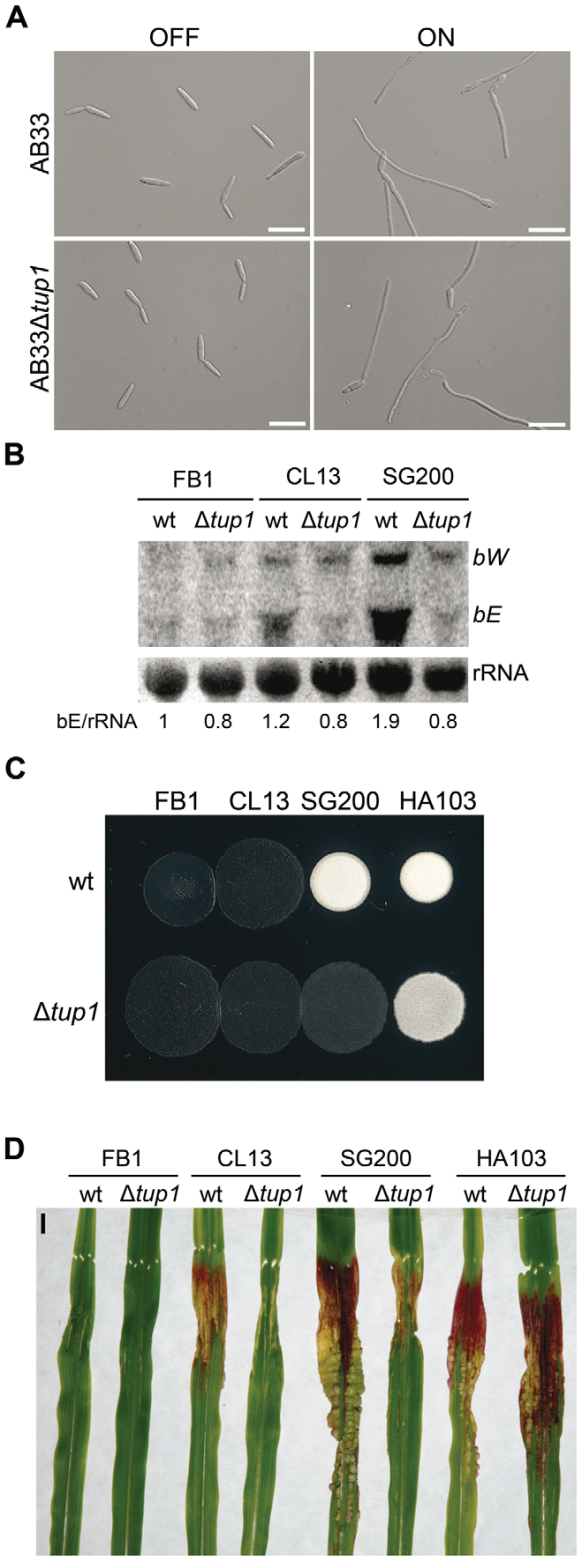
Genetic interaction between *tup1* and the *b* mating-type locus. (A) Induction of *b*-compatible heterodimer in the AB33 background. Expression of *bE* and *bW* genes was induced by a shift from ammonium (OFF) to nitrate (ON) containing minimal media. *b*-dependent filament formation could be observed both in wild-type and *tup1* mutant strains. Pictures were taken 5 hours post-induction. Scale bars represent 20 µm. (B) *b*-gene expression level in wild-type and *tup1* deletion strains of CL13 (*a1 bE1/bW2*), SG200 (*a1 mfa2 bE1/bW2*) and HA103 (a1 (bE1/bW2)^con^). 10 µg of total RNA extracted from each strain grown on charcoal minimal media for 48 hours at 25°C was loaded per lane. Methylene blue stained rRNA was used as loading control. Numbers indicate the relative signal of *bE* gene in regard to rRNA. (C) Filamentation capacity of the indicated strains growing on PD-charcoal plates during 24 hours at 25°C. White fuzzy colonies indicates *b*-dependent filament formation. (D) Representative images showing the most prevalent disease category for wild-type and *tup1* mutant infected plants. Strains are indicated below. The FB1 (*a1 bE1/bW1*) background, which harbors an incompatible *b*-heterodimer, was used as control. Scale bar represent 1 cm.

As *tup1* mutants are unable to form dikaryotic hyphae at wild-type levels, we wondered whether *tup1* regulates genes downstream of the *b* locus, thus compromising the fungal dimorphic transition in *tup1* mutants. To this end, we used the AB33 strain in which expression of a compatible bE1/bW2 heterodimer is under the control of the *nar* inducible promoter [62]. When this strain is grown in inducing conditions it forms a *b*-dependent filament. We found that deletion of *tup1* in this background did not affect its filamentation capacity (Figure 6A; see Figure S6 for quantification). This result suggests that Tup1 is affecting processes upstream of the *b* locus or, alternatively, is acting on a parallel pathway regulating filamentation. To discern between these two possibilities, we extracted total RNA from SG200 and SG200Δ*tup1* fungi grown on charcoal-containing media for 48 hours and quantified the expression of *bE* and *bW* by Northern blot. We observed a strong decrease in both gene transcripts in SG200Δ*tup1* indicating that *tup1* is required for the normal expression of *b* loci genes (Figure 6B lanes 5 and 6).

To test if constitutive *b* expression could rescue the filamentation and virulence phenotypes of *tup1* mutants, we took advantage of the HA103 strain, which harbors a compatible bE1/bW2 heterodimer under the control of constitutive promoters [52]. Deletion of *tup1* in HA103 did not produce the filamentation and virulence defects described for the SG200 background (Figure 6C, 6D and Figure S7), indicating that constitutive *b* expression partially rescues these phenotypes. To better understand the effect of *b* expression on the *tup1* mutant virulence phenotype, we used the HA103 parental strain, CL13 [59], which carries compatible *bE1 and bW2* genes under the control of their own promoter and lacks the constitutively-expressed *mfa2* gene present in SG200. Deletion of *tup1* from CL13 led to a 90% reduction in maize tumor formation (Figure 6D and Figure S7), revealing an even clearer *b*-genes dependent rescue of *tup1* mutant phenotypes. Interestingly, the expression level of the *b* genes correlated with the phenotype of the wild-type and Δ*tup1* strains (Figure 6B). Moreover, when we focused on the CL13 and SG200 backgrounds, we observed that the SG200Δ*tup1* strain had a *b* expression level, filamentation and virulence capacity comparable to the wild-type CL13 strain (Figure 6, Figure S7 and Figure S8). Thus, the effect of deleting *tup1* from SG200 seems to be equivalent to removing its constitutive expression of *mfa2*, which would suggest a putative role for the pheromone responsive pathways in *tup1* mutant phenotypes.

### Tup1 is required for *mfa1* gene expression, and conjugation tube formation upon pheromone stimulation

In our earlier experiment we bypassed the requirement for cell fusion by using the SG200 strain to identify a post-fusion requirement for *tup1* in *U.maydis* filamentation. However, this experiment does not exclude a role for *tup1* in mating between compatible strains as well, especially since both *a* and *b* loci genes are in the same position of the genetic pathway that controls the dimorphic transition. Moreover, as commented above, the similarity between SG200Δ*tup1* and CL13 strains may reflect a role for *tup1* in the transduction of the pheromone signal.

**Figure 7:**
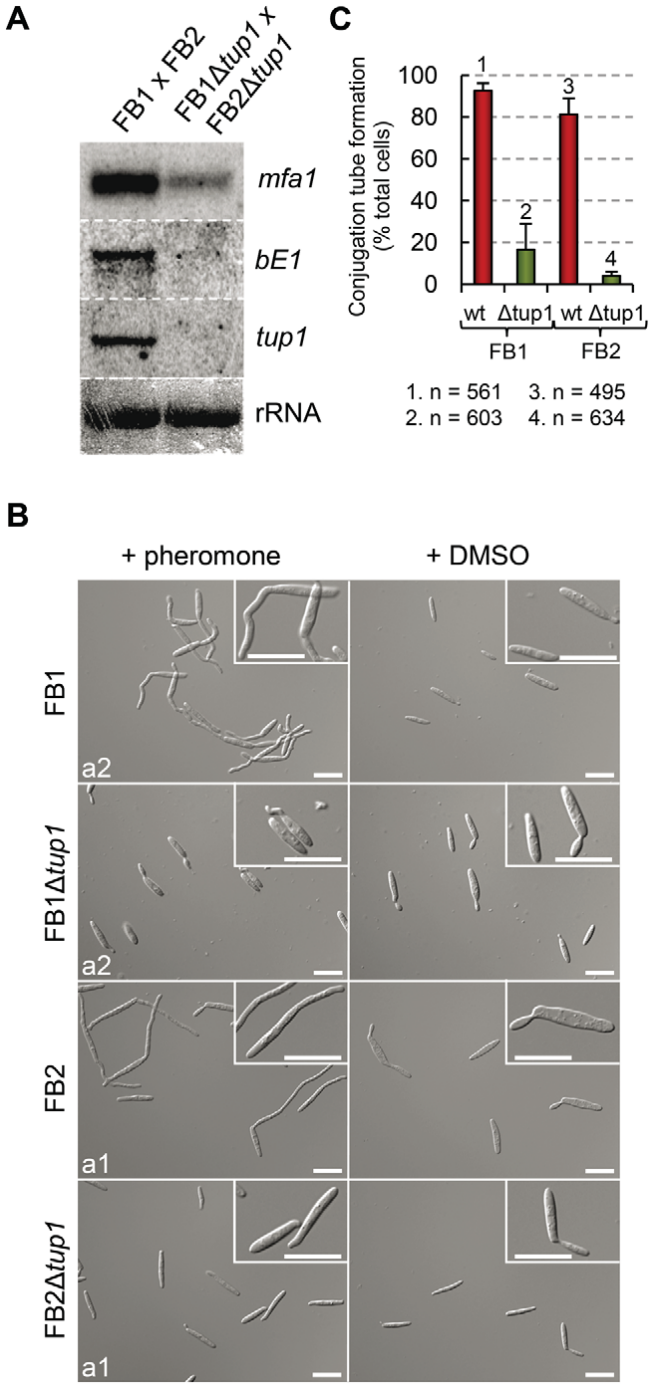
Pheromone response and conjugation tube formation in *tup1* mutants. (A) Expression level of *mfa1* and *bE1* in FB1 vs FB2 and FB1Δ*tup1* vs FB2Δ*tup1* crosses after 24 h on charcoal containing plates. *tup1* expression was used as experimental control and rRNA used as loading control. (B) DIC images of conjugation hyphae in wild-type and *tup1* mutant strains. Wild-type and *tup1* mutant strains were grown on CM liquid media until exponential phase and then exposed to the pheromone of the opposite mating type or DMSO (pheromone solvent) for 5 hours. Strains (left), and pheromone or DMSO treatments (top) are indicated. Type of pheromone (a1 or a2) is shown inside each picture. Scale bars indicate 20 µm. (C) Quantification of conjugation hyphae formation in wild-type and *tup1* deletion strains. Total number of cells counted is indicated below the chart. Mean values of three independent experiments and the standard deviation are shown.

To test this possibility, we extracted total RNA from a FB1Δ*tup1* vs FB2Δ*tup1* cross grown on charcoal-containing media for 24 hours and compared *mfa1* and *bE1* expression with a wild-type strains cross by Northern blot. In the wild-type cross, as a result of the recognition of pheromones by receptors of opposite mating types, activation of pheromone responsive pathways takes places, which is reflected in the expression of genes at both *a* and *b* loci. In the case of the *tup1* mutant cross, however, we observed reduced *mfa1* and *bE1* expression (Figure 7A), indicating that *tup1* is necessary for wild-type expression of these genes. Accordingly, FB1Δ*tup1* and FB2Δ*tup1* strains drastically reduced conjugation hyphae formation upon stimulation with synthetic pheromones of the opposite mating type (Figure 7B and 7C).

Thus, *tup1* is required for signal transduction upon stimulation with pheromone and expression of genes at both *a* and *b* loci, which is reflected in the observed pre and post-fusion defects of Δ*tup1* cells.

### Tup1 controls *a* and *b* loci genes downstream of the MAP kinase cascade

**Figure 8:**
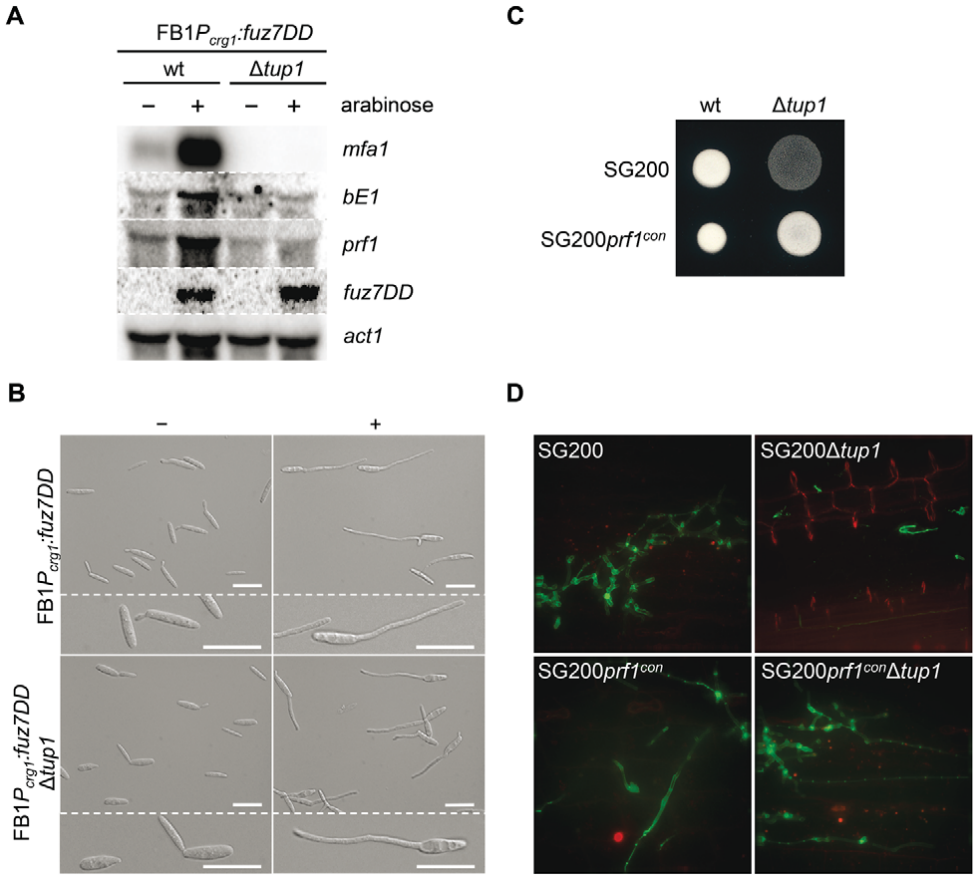
Tup1-dependent regulation of mating-type genes and *prf1* transcription factor. (A) *mfa1*, *bE1*, and *prf1* expression levels upon *fuz7DD* allele induction. Expression of the *fuz7DD* allele was induced by a shift from a glucose to arabinose containing CM media. Total RNA was extracted 5 hours post-induction and 10 µg were loaded in each lane. *U. maydis* actin was used as loading control. Strains (above) and probes (right) are indicated. (B) Conjugation tube formation upon *fuz7DD* induction. Pictures were obtained by optical microscopy 5 hours post-induction. - (glucose) and + (arabinose) indicate non-inducing and inducing conditions, respectively. Scale bars represent 20 µm. (C) Filamentation of constitutive expressed *prf1* strains on charcoal media. SG200, SG200*prf1^con^* and their derivatives were spotted alone on charcoal plates and grown at 25°C for 24 hours. White fuzzy colonies appearance indicates formation of filaments. (D) *On planta* filamentation of constitutive expressed *prf1* strains. SG200, SG200*prf1^con^* and their derivatives were inoculated into maize plants and their filamentation capacity was determined 24 hours post-inoculation.

The expression of *a* and *b* loci genes is controlled by the cAMP and MAP kinase pathways through their common effector Prf1. To situate *tup*1 within this genetic context, we used the FB1*P_crg1_:fuz7DD* strain, which harbors a constitutively active allele of *fuz7* MAPKK under the control of the arabinose inducible promoter *crg1* [51] (see Figure 1 for components of the MAP kinase pathway). Upon induction, this strain promotes the expression of *a* and *b* loci genes via the Prf1 transcription factor. After deleting *tup1* from this strain, we checked for *a* and *b* loci gene expression under inducing conditions. As expected, increased transcription for genes at both loci was observed in the wild-type strain; however, this was not the case for the *tup1* mutant, indicating that Tup1 regulates *a* and *b* gene expression downstream of Fuz7 MAPK kinase (Figure 8A). Since Tup1 is involved in regulating the expression of genes related to glucose metabolism, the expression level of Fuz7 under the control of the *crg1* promoter was also examined. No difference in *fuz7DD* expression was observed between the wild-type and the Δ*tup1* strains (Figure 8A).

Apart from its effect on the expression of the previously mentioned genes, induction of the *fuz7DD* allele, promotes conjugation tube formation through a Prf1 independent pathway that also requires the action of Kpp2 MAP kinase [51]. Thus, we wondered whether the induction of *fuz7DD* in the *tup1* deletion strain could also induce conjugation tube formation. As shown in Figure 8B, *tup1* mutants in this background were able to form conjugation hyphae at similar levels to wild-type fungi in inducing conditions (Figure S9 for quantification). This result makes it unlikely that Tup1 is regulating conjugation tube formation downstream of the MAP kinase cascade and, at the same time, strongly suggest that *tup1* regulates mating-type genes downstream of Kpp2 MAP kinase.

### Tup1 is required for expression of *prf1* transcription factor

We have shown that *tup1* seems to regulate the expression level of *a* and *b* loci genes acting downstream of the MAP kinase cascade. Since the Prf1 transcription factor is the genetic element connecting the MAP kinase cascade and the mating-type genes, we measured *prf1* expression level in a FB1*P_crg1_:fuz7DD* background under inducing conditions. The removal of *tup1* prevented the increase in *prf1* expression (Figure 8A), indicating that *tup1* is required for *prf1* expression upon MAP kinase cascade induction. Moreover, the filamentation defects on charcoal-containing media as well as on the plant surface were rescued with the constitutive expression of *prf1* (Figure 8C and 8D). These results strongly suggest that *tup1* affects mating and *b*-dependent filament formation through control of *prf1* transcription factor expression level rather than by controlling the expression of *a* and *b* loci genes directly.

### *Tup1* deletion affects the expression of several *b* and pheromone/*fuz7DD* regulated genes as well as the *rop1* transcription factor

**Figure 9:**
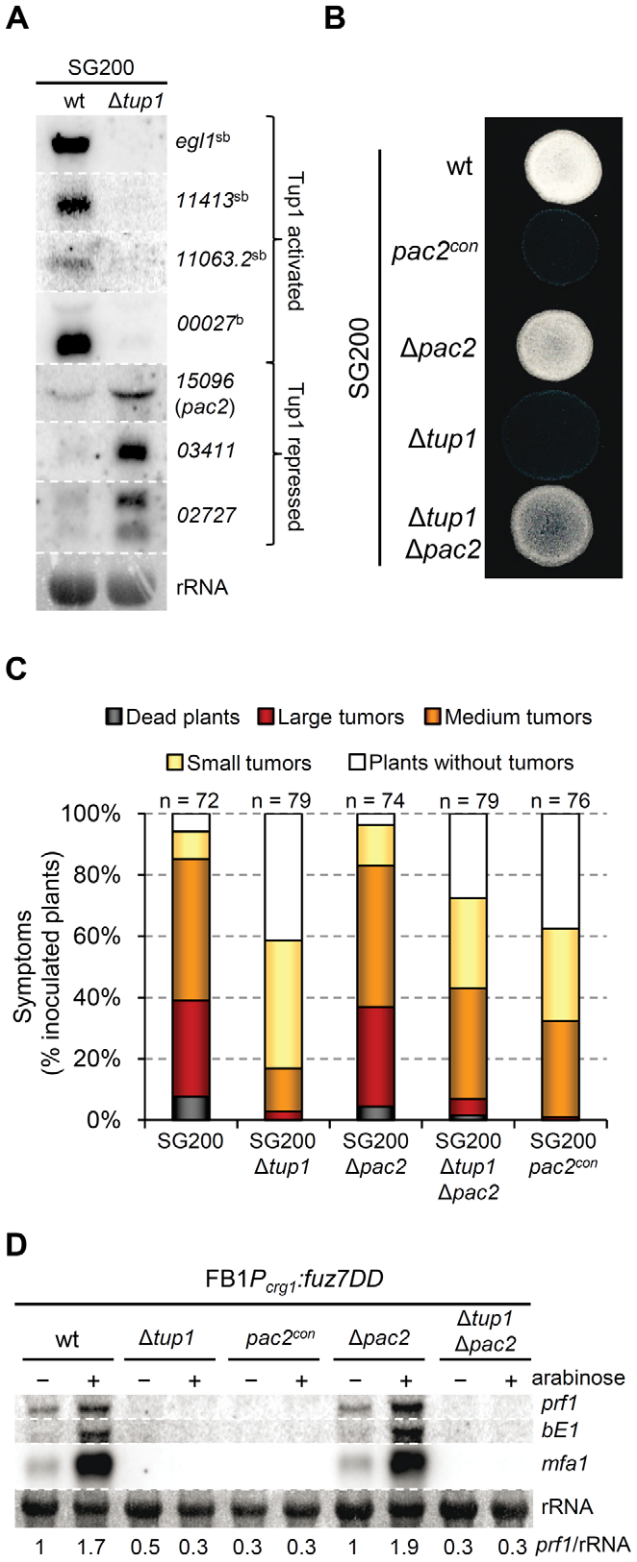
Microarray validation and *pac2* mutants filamentation and virulence phenotypes. (A) Validation of microarray data by Northern blot. Probes (right) and strains (top) used are indicated. *b*-dependent (b) and strictly *b*-dependent genes (sb) according to [63], are indicated. Methylene blue stained rRNA was used as loading control. Total RNA was extracted from the indicated strains growing on minimal media charcoal-array medium for 48 hours at 25°C. A total of 10 µg of RNA was loaded per lane. (B) Filamentation capacity of the *pac2* mutant strains. Strains indicated (left) were spotted alone on PD-charcoal plates and grown for 24 hours at 25°C. *pac2* ^con^ indicates constitutive expression of *pac2* from the *otef* promoter. (C) Pathogenicity of *pac2* mutant strains. Seven day old maize seedlings were infected with the indicated strains (color legend). Total number of infected plants (n) is indicated above each column. Symptoms were scored 14 dpi. Tumors categories correspond to: large tumors (>5 mm), medium tumors (1–5 mm) and small tumors (<1 mm). Represented are the main values of three independent experiments. (D) Pac2-dependent regulation of *prf1*. *Prf1* expression level of the indicated strains upon *fuz7DD* allele induction was measured by Northern blot. Expression of the *fuz7DD* allele was induced by a shift from a glucose (-) to arabinose (+) containing CM media. 10 µg of total RNA were loaded per lane. rRNA was used as loading control. Numbers indicate the relative signal of *prf1* gene in regard to rRNA.

As constitutive *bE/bW* expression did not fully complement Δ*tup1* phenotypes, we were interested in identifying other Tup1 regulated genes, that might also have roles in the dimorphic transition and virulence in *U. maydis*. For this purpose we performed a microarray analysis with custom Affimetrix array (MPIUstilagoA), covering 5823 of the 6787 predicted *U. maydis* genes, and compared the gene expression of SG200 and SG200Δ*tup1* strains grown on MM-charcoal array plates for 48 hours (see Methods). We identified a total of 115 genes (around 2 % of the covered genes) with altered expression in the *tup1* mutant strain. Of these, 59 were upregulated and 56 downregulated. Within this list appear the *bE* and *bW* genes together with 34 genes that have also been described as *b* regulated genes [63], and 17 genes described as pheromone regulated [64] (Table S3). Thus, around 36% of the genes directly or indirectly regulated by *tup1* are also regulated upon *bE/bW* heterodimer and/or pheromone/*fuz7DD* induction, in agreement with our earlier results and supporting the quality of our dataset. Additionally, in order to experimentally validate our microarray data, the differential expression of some of the genes was confirmed by Northern blot analysis (Figure 9A).

All the 115 Tup1-regulated genes were classified in functional categories using the Blast2Go tool [65]. Enrichment analysis of genes up-regulated by the deletion of *tup1* did not reveal a significant over-representation in any of the GO categories (Table S4). Of the genes down-regulated upon *tup1* deletion our analysis revealed a significant over-representation in two GO categories: “Carbohydrate metabolic process” (GO:0005975; 8 genes) and “Antioxidant activity” (GO:0016209; 3 genes) (Table S4). 4 of the 8 genes belonging to the first category were also *b*-regulated genes, with two of them defined as strictly *b*-dependent (Table S3). The second category comprises proteins involved in the inhibition of dioxygen or peroxide-induced reactions and could be related to pathogenicity since production of these compounds is a well-characterized plant defense mechanism [66], [67], and H_2_O_2_ detoxification is required for *U. maydis* virulence [68].

Interestingly, several *tup1*-regulated genes are associated with processes that could be related to the morphological switch from yeast-like to filamentous growth. Almost 10% of these genes are potentially involved in cell wall synthesis or modification, revealing that the altered yeast-to-hypha transition, promoted by deletion of *tup1,* results in a different cell wall composition.

Significantly, we found that *rop1*, that encodes a direct activator of Prf1, was down-regulated in the *tup1* deletion strain (Table S3). This suggests an indirect role for *tup1* in controlling *prf1* expression. Rop1 has been described as being required for the mating of compatible strains on charcoal containing media, with a post-fusion role, due to the inability of SG200Δ*rop1* to form white fuzzy colonies on charcoal plates. It is essential for conjugation tube formation upon pheromone stimulation, and for expression of pheromone-responsive genes [61]. These phenotypes clearly resemble the situation described for *tup1* mutants; however, *rop1* mutants are fully pathogenic, with no mating or filamentation defects described on the plant leaf surface [61].

In addition to *rop1*, we identified an interesting candidate gene, *um15096*, that could be related to the *tup1* mutant phenotypes. In *Schizosaccharomyces pombe*, a homologue of *um15096*, named *pac2*, has been shown to be a repressor of *ste11* (the putative functional homologue of *prf1*) [69]. Interestingly, *um15096/pac2*, herein referred to as *pac2*, appeared over-expressed in the *tup1* deletion strain. To check whether this putative *prf1* repressor could also be playing a role during filamentation and pathogenic development, we over-expressed *pac2* by integrating an extra copy of the gene under the control of the *otef* constitutive promoter in the *ip* locus of the SG200 strain. Filament formation of SG200*pac2* ^con^ was reduced on charcoal containing media (Figure 9B) and, more importantly, pathogenicity was reduced to levels comparable to *tup1* mutants (Figure 9C). The fact that *pac2* is over-expressed in *tup1* mutants together with the observation that ectopic *pac2* expression decreases filamentation and virulence in the wild-type strain, strongly suggest that *pac2* expression contributes to the filament formation and pathogenic defects of Δ*tup1* cells. Consistent with this, the deletion of *pac2* from SG200 resulted in wild-type filamentation and infection rates (Figure 9C). When *prf1* expression was induced by constitutively activating the MAPK pathway at Fuz7 level, overexpression of *pac2* abolished its expression, while deletion of *pac2* did not apparently affect it. Similar results were observed for *mfa1* and *bE1* genes. The double Δ*tup1*Δ*pac2* mutant showed the same level of expression as the single Δ*tup1* strain (Figure 9D); probably as consequence of the regulation of *rop1* via Tup1. Surprisingly, *pac2* deletion, weakly restored the filamentation and infection defects shown by SG200Δ*tup1* strain (Figure 9B and 9C), indicating that Pac2 contributes to *tup1* deletion strain phenotypes.

In summary, our microarray data reveal that at least 36% of the genes whose expression is affected by deletion of *tup1* seems to be a consequence of *tup1*-dependent regulation of *a* and *b* loci genes through *prf1*. Moreover, the role of Tup1 in the control of *prf1* expression could be explained by the altered expression of *rop1* and *pac2* observed in the *tup1* mutant strain.

### Tup1 affects the expression of the *prf1* transcriptional regulators *rop1* and *hap2* but not *crk1*

**Figure 10:**
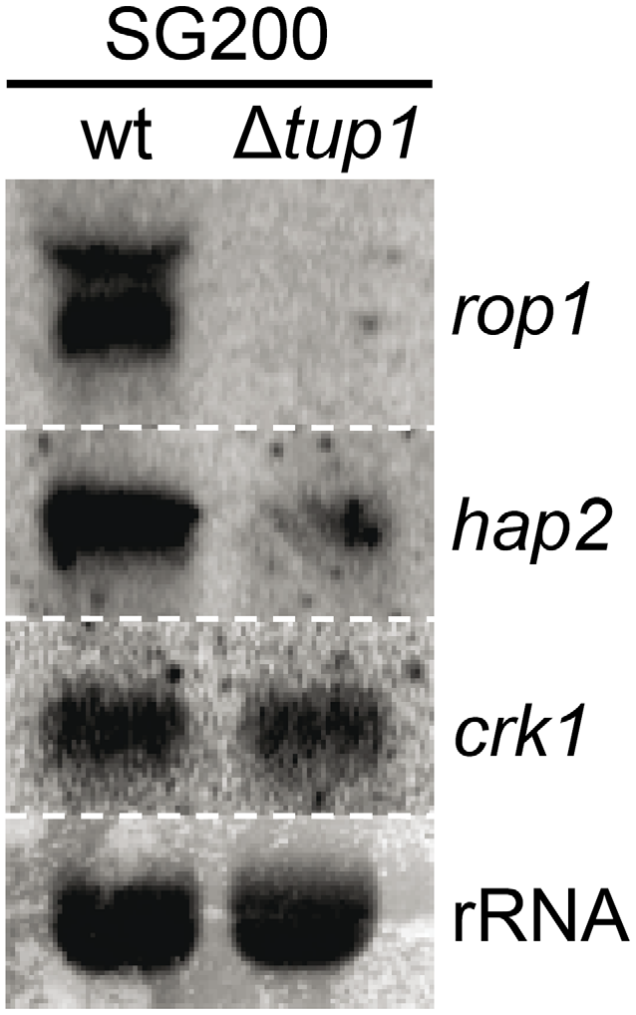
*tup1* is required for wild-type expression levels of the *prf1* transcriptional regulators *rop1* and *hap2*. Northern blot of *rop1*, *hap2* and *crk1*. 10 µg of total RNA extracted from the indicated strains growing on minimal media charcoal plates for 48 hours at 25°C was loaded per lane. rRNA was used as loading control.

As Tup1 seems to have an indirect effect on *prf1* transcription level through Rop1 and, putatively, Pac2, we wondered whether the expression of other known *prf1* regulators could be affected in *tup1* deletion strains. Apart from Rop1, *prf1* is known to be directly regulated by Hap2 [70] and indirectly through the MAP kinase Crk1 [71]. Northern blot assays of SG200 and SG200Δ*tup1* grown on charcoal media showed that the expression level of *crk1* was unaffected in *tup1* deleted strain. In contrast, the levels of *rop1* and *hap2* were reduced in comparison to the wild-type strain (Figure 10). However, as Crk1 acts on *prf1* indirectly, and since it has been previously reported that the effect of Crk1 on *prf1* depends on the *prf1* promoter UAS [71], we tested whether Tup1 could regulate *prf1* via its UAS. For this purpose, we used the HA232 strain, which harbors a GFP reporter gene under the control of the *prf1* promoter UAS (see [53] for details). In this strain, GFP is strongly expressed when grown on glucose-containing media, while its expression is reduced on a maltose containing media [53]. As is shown in Figure S10, the expression levels of the reporter gene were indistinguishable in Δ*tup1* mutants from the wild-type in all the conditions tested. This indicates that Tup1 is unlikely to act via the *prf1* promoter UAS, in contrast to Crk1. Thus, the effect of Tup1 on *prf1* expression seems to be mediated via Rop1 and Hap2 but not through the Crk1 pathway.

To sum up, although other factors may be implicated in *tup1* mutant phenotypes, Tup1 seems to control the dimorphic transition and participates in the virulence program of *U. maydis* by indirectly regulating *prf1* expression via altered *rop1* and *hap2* expression levels, and possibly also through *pac2*, which would lead to a down-regulation of *prf1*-dependent expression of *a* and *b* loci genes and their related phenotypes.

## Discussion

In the basidiomycete phytopathogen *U. maydis*, the switch from non-infective yeast-like growth to an infective filament formation occurs in response to different environmental cues, and is tightly controlled by complex genetic pathways in order to ensure the coordination and timing of the different processes associated with dimorphism. In this work, we have shown that the highly conserved general transcriptional repressor Tup1 plays a central role in controlling the proper expression of the genes implicated in the genetic control of mating, filamentation, and pathogenic development of this corn smut fungus.

Tup1 has been shown to be important during growth of vegetative cells in other fungi such as *S. cerevisiae*, *C. neoformans* or *P. marneffei* [12], [27], [72]. In the case of *Ustilago maydis*, differences could be observed in the *tup1* mutants, although none of these were statistically significant. Interestingly the normal growth of Δ*tup1* strains contrasts with the poor growth capacity described for *U. maydis* strains harboring a partial deletion of *sql1*, the functional homolog to *S. cerevisiae SSN6*. However because these strains were not stable, the role of Sql1 could not be completely analyzed [34]. Thus a comparison between Tup1 and Sql1 of their growth capacity on *U. maydis* vegetative cells cannot be properly established. In other fungi, single deletions of *tup1* and *ssn6* have been reported to result in different phenotypes [73]-[76]. For example, the deletion of *SSN6* but not of *TUP1* homologues is lethal in *S. pombe* [75] and *Aspergillus nidulans* [76]. Moreover, Tup1 and Ssn6 have been shown to regulate different set of genes [74] and to form independent complexes in *C. albicans* [77].

A central question in this study was whether *tup1* is involved in the infectious process of plant pathogenic fungi. We have observed that infections with Δ*tup1* cells lead to a reduction in tumor formation, plant death, and a failure of spore formation, indicating that Tup1 is required for full pathogenic development in *U. maydis,* and making *tup1* mutants unlikely to cause damage in natural environments. Thus, *tup1* seems to play a conserved role in virulence of animal and plant fungal pathogens.

The next key question was to try to understand the mechanism by which *tup1* is required for normal tumor formation. Our results suggest that the virulence phenotype of Δ*tup1* cells has two main causes: (i) a recognition problem between compatible partners, due to the inability of *tup1* mutants to form conjugation hyphae upon pheromone stimulation, and (ii) a filamentation defect, due to the inability of SG200 to form filaments at wild-type levels both on PD-charcoal plates and on the plant leaf surface. Additionally, the fact that the differences on conjugation hypha formation between FB1Δ*tup1* and FB2Δ*tup1* strains, though not statistically significant, together with the differential filamentation showed by crosses of these strains with their respective compatible wild-type strains on charcoal plates, suggest also a role for Tup1 in cell fusion, at least in the FB2 background. These defects result in *tup1* mutants being unable to properly undergo dimorphic transition. These findings suggest that the impaired pathogenicity of *tup1* mutant animal and plant fungi may also depend on a conserved role in the yeast-to-hypha transition.

Consistent with the conjugation and filamentation phenotypes of *tup1* mutants, the expression of *a* and *b* loci mating-type genes was reduced in *tup1* deletion strains, most likely as a consequence of Tup1-dependent regulation of the *prf1* transcription factor. Microarray analysis of SG200Δ*tup1* during filamentation on charcoal media revealed a number of mis-regulated genes whose expression was also affected upon *b*-compatible heterodimer and/or pheromone/*fuz7DD* induction, including the *b* locus genes themselves, supporting the proposed role for *tup1* during *U. maydis* mating and dikaryotic filament formation. On the other hand, in our microarray analysis we did not detect *tup1*-dependent changes in gene expression for any of the *b-*dependent genes previously described as being essential for pathogenicity [60], [63], [78], which is consistent with the ability, albeit reduced, of *tup1* mutants to induce tumors in maize.

Interestingly, the main effector that links *tup1* to the control of dimorphism seems to be conserved between *U. maydis* and *C. albicans.* In contrast, the genetic pathways by which *tup1* acts on filamentation seem to differ, depending on the genetic control of hypha-specific genes in each organism. In *C. albicans*, Tup1 is proposed to control filamentous growth through the repression of hypha-specific genes by forming complexes with the transcriptional repressors Rfg1 and Nrg1, rather than affecting the elements in the Cph1-mediated MAPK and Efg1-mediated cAMP pathways [7], [54]–[56]. Moreover, expression analysis of filament-specific genes in Δ*cph1/*Δ*cph1*, Δ*efg1/*Δ*efg1* and Δ*tup1/*Δ*tup1* strains revealed common and divergent target genes [7]. Thus, Tup1 integrates into the network system proposed for the control of filament-specific genes in this fungus [7], [10]. On the other hand, in *U. maydis,* Tup1 controls infective filament-specific gene expression via a central regulatory, the Prf1 transcription factor, which is transcriptionally and post-translationally regulated by the cAMP and MAPK pathways [47], [51]–[53]. Interestingly, *U maydis* Prf1 is a High Mobility Group (HMG) transcription factor, similar to *C. albicans* Rfg1. Thus, an analogous mechanism, implicating a Tup1-Prf1 complex, could explain the roles of Tup1 in the regulation of hypha specific genes in *U. maydis*. Moreover, in *S. cerevisiae*, a complex between Tup1p and the HMG-transcription factor Rox1p has also been proposed [19], [79]-[81]. *S. cerevisiae ROX1*, whose deletion can be complemented by *C. albicans RFG1* [33], is known to control hypoxic gene expression in a *TUP1* dependent manner [19], [79]–[81]. Additionally, the deletion of *TUP1* increases the expression of *ROX1* [82], [83], but Rox1p itself is also able to regulate its own expression [83]. In aerobic conditions these observations can be explained by the proposed Tup1p-Ssn6p-Rox1p complex which would regulate *ROX1* expression and Rox1p-dependent hypoxic gene expression. In anaerobic conditions, however, the regulation of *ROX1* expression seems to implicate an anaerobic repressor that requires Tup1p for its function [83]. Similarly, in *U. maydis*, the expression of *prf1* is dependent on Tup1 and *prf1* is also self-regulated [52]. However, when we analyzed the effect of Tup1 on *prf1* expression level more deeply, we observed that at least two direct activators of Prf1 were also down-regulated upon *tup1* deletion, *rop1* and *hap2*. This finding, although not excluding a putative Tup1-Prf1 complex, points to an indirect effect of Tup1 on the expression of *prf1* and its regulated genes. *Rop1* is required for pheromone response and for fuzzy colony formation on charcoal-containing plates, but is dispensable for mating and filamentation on the plant leaf surface. In the case of *hap2*, it is known to be essential for the pheromone response and has also an effect on the filamentation capacity of SG200 that seem to be conserved *on planta*. Thus, we propose that the effect of Tup1 on *prf1* is the sum of the effects of Tup1 in both *rop1* and *hap2* on artificial media, while only the effect on *hap2* would be responsible for the *on planta* phenotypes. The drastic effect of *tup1* deletion on *prf1* expression levels on charcoal plates may be diminished on the plant leaf surface as *rop1* is dispensable in this situation.

**Figure 11:**
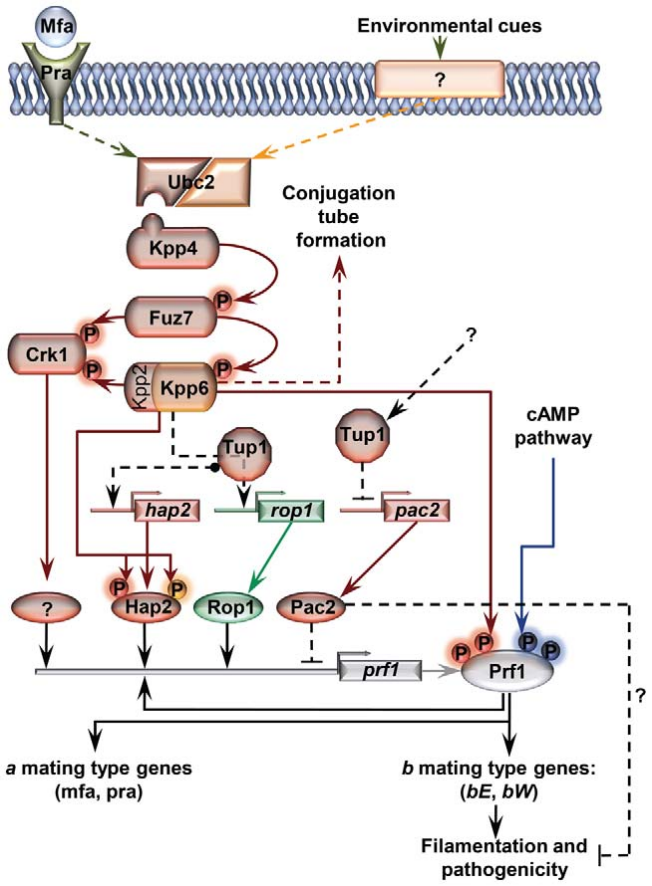
Proposed model for the roles of Tup1 in the control of mating-type genes. The MAP kinase pathway is shown in red. Black arrows represent transcriptional control. Components exclusively required in laboratory conditions (charcoal, pheromone stimulation, etc) are shown in green. Components specifically required during pathogenesis are shown in orange. In laboratory conditions the effect of Tup1 on *prf1* expression would be mediated via its control of *hap2*, *rop1* and *pac2* expression levels. During infection, where *rop1* is not required, Tup1 would control *prf1* expression through *hap2* and *pac2*. Question marks indicate putative elements or interactions.

In this work, we have also described a new gene, *pac2*, which is likely to be playing a role in the *tup1* mutant virulence phenotype, since its over-expression causes a decrease in the pathogenic capacity of *U. maydis* SG200 strain and its expression is increased in the SG200Δ*tup1* strain. Since the homologue of this gene in *S. pombe* is a repressor of *ste11* [69], the putative functional homologue of *prf1,* we analyzed the relationship between Pac2 and Prf1 in *U. maydis*. We found that over-expression of *pac2* in a FB1*P_crg1_:fuz7DD* strain abolished the *prf1* expression observed in the wild type strain establishing Pac2 as a repressor of Prf1. Accordingly, the deletion of *pac2* in a SG200Δ*tup1* strain partially restored its filamentation and virulence defects. However, the double Δ*tup1*Δ*pac2* mutant in the FB1*P_crg1_:fuz7DD* background shows the same *prf1* expression level than the single Δ*tup1* strain, probably because of Tup1 control of *rop1* and *hap2*. Nevertheless since *prf1* regulation on charcoal plates or during virulence integrates several imputs besides the MAPK pathway the relationship between *pac2* and *prf1* in the regulation of filamentation and pathogenicity cannot be fully established. Thus, the final role of *tup1* in *U. maydis* virulence is also likely to be linked to its control of *hap2* and *pac2* mRNA levels (Figure 11).

Surprisingly, although Tup1 is described as a general transcriptional repressor, the deletion of *tup1* from *U. maydis* leads to the down-regulation of the genes that control the dimorphic transition, suggesting an activator role for *tup1* in controlling them. On the other hand, determining how Pac2 controls *prf1* gene expression would help to determine the role of *tup1* as an activator and/or repressor during dimorphism. The way Tup1 seems to control the expression of the *prf1* transcription factor, through *hap2* and *rop1* and, putatively, *pac2*, clearly reflects the complex genetic regulation that *prf1*-related processes require.

Similarly, the number of genes that we found to be up- or down-regulated following *tup1* deletion when cultured on charcoal-containing media was equivalent. Thus, under the conditions tested, the loss of *tup1* causes a similar effect on both the de-repression and repression of genes. Although this could reflect indirect changes in genes expression resulting from the repression of Tup1-gene targets, it is nevertheless an intriguing observation. Regarding an activating role for Tup1, previous studies have also shown that Tup1 can behave as an activator as well as a repressor of the same target gene in different conditions [84] or different genetic backgrounds [85] in *S. cerevisiae*.

Finally, we have shown that *tup1* seems to be required for spore production inside maize tumors. Roles for Tup1 in sporulation have been previously reported in other fungi. In *S. cerevisiae*, the sporulation-specific genes *DIT1* and *DIT2*, which are required for spore wall formation, are regulated by Tup1p [86]; in *Neurospora crassa*, mutants for *rco-1*, the homologue of *TUP1*, are aconidial [87]; and in *C. neoformans*, *tup1* deletion considerably reduces spore production [27].

In summary, our work provides new insights into the complex regulatory circuits for sexual and pathogenic development of *U. maydis*. We have identified for the first time a requirement for *tup1* at several steps of the life cycle of a pathogenic plant fungus, including in the genetic pathways controlling dimorphism and virulence. Our findings contribute to a better understanding of the role of this general transcriptional repressor in pathogenic fungi and of the precise genetic control that these pathogenesis-related processes require. We consider that the roles and mechanisms of action described for *U. maydis tup1* in this work will also be extremely valuable for studying the roles of *tup1* in the transcriptional regulation of morphogenetic processes in other organisms.

## Methods

### Strains, growth conditions and plasmids

*Escherichia coli* DH5α was used for cloning purposes. Growth conditions for *E. coli* [88] and *U. maydis* [42], [89] and the quantification of appressoria formation on the plant leaf surface [60] have been described previously. Quantification of filaments was performed as for the appressoria. For studies of growth rates and morphology, cells were grown on YEPSL liquid media for 12 hours, then diluted in the same media to an OD_600_ of 0.05 and grown until an OD_600_ of 0.8-1. Exponential growth cultures were examined under the microscope and transferred to solid plates for colony morphology studies. Growth rates on liquid media were determined by counting cells at different time-points. For charcoal mating and filamentation assays, cells were grown on YEPSL until exponential phase, washed twice with water, spotted onto PD-charcoal plates and grown for 24–48 hours at 25°C. For charcoal-grown cells used for RNA extractions, cells were spread out on charcoal plates at a concentration of OD_600_  = 0.1 per cm^2^. For DNA array charcoal media see below. *U. maydis* strains relevant to this study are listed in Table S5. Induction of *nar* promoter in AB33 [62] and *crg* promoter in FB1*P_crg1_:fuz7DD* [51] strains, and their derivatives, were done as previously described. Mating assays were performed as previously described in [90]. Pheromone stimulation was performed following the protocol of [51]. For pathogenicity assays, *U. maydis* strains were grown to exponential phase and concentrated to an OD_600_ of 3, washed twice in water, and injected into 7 days old maize (*Zea mays*) seedlings (Early Golden Bantam). Tumor formation was quantified 14 to 21 days post infection. Data are expressed as means ±SD of triplicate samples. Statistical significance was assessed using Statistical Calculators (http://www.graphpad.com/quickcalcs/index.cfm) and considered significant if p values were <0.05.

### DNA and RNA procedures

Molecular biology techniques were used as described by [88]. *U. maydis* DNA isolation and transformation procedures were carried out following the protocol of [91]. Deletion constructs were generated according to [36]. To generate single deletion *U. maydis* mutants for *tup1* (Um03280), *pac2* (Um15096) and *um04807* genes, fragments of the 5′ and 3′ flanks of their open reading frames were generated by PCR on *U. maydis* FB1 genomic DNA with the following primer combinations: UmTUP1KO5-1/UmTUP1KO5-2 and UmTUP1KO3-1/UmTUP1KO3-2; UmPAC2KO5-1/UmPAC2KO5-2 and UmPAC2KO3-1/UmPAC2KO3-2; Um04807KO5-1/Um04807KO5-2 and Um04807KO3-1/Um048071KO3-2; (Sequences in Table S2). These fragments were digested with *Sfi*I and ligated with the 1.9 Kb *Sfi*I carboxin resistance cassette, 2.7 Kb *Sfi*I hygromycin resistance cassette, or 1.5 Kb *Sfi*I neourseotricin resistance cassette as described previously [35]. Ligation products were then clone into pGEM-T-EASY vector (Promega). PCR generated linear DNA for each construct was used for *U. maydis* transformation.

For complementation of the *tup1* deletion, the p123-*tup1* plasmid was generated. p123-*tup1* is a p123 [92] derivative in which the eGFP fragment has been substituted with the *tup1* open reading frame . For this purpose, the *tup1* open reading frame was amplified by PCR with the oligonucleotides Tup1-Start and Tup1-Stop, which contain *Nco*I and *Not*I restriction sequences respectively. Phusion high fidelity DNA polymerase (Invitrogen) was used. The PCR product was digested with *Nco*I and *Not*I, purified, and cloned into a p123 vector digested with the same restriction enzymes. Positive cloning was verified by restriction analysis and sequencing. To generate SG200Δ*tup1P_otef_:tup1* strain, p123-*tup1* was linearized with *Ssp*I and integrated into SG200Δ*tup1 ip* locus by homologous recombination.

For over-expression of *pac2*, the p123-*pac2* plasmid was generated by replacing the eGFP fragment from p123 with the *pac2* open reading frame. The Pac2 open reading frame was amplified using the oligonucleotides UmPac2ATGSmaXma y UmPac2StopNotI, digested with *Xma*I and *Not*I restriction enzymes and ligated into the p123 vector digested with the same enzymes. Successful cloning was verified by restriction analysis and sequencing. To generate SG200*pac2* ^con^, p123-*pac2* was linearized with *Ssp*I and integrated into SG200 wild-type strain *ip* locus.

For constitutive expression of *pac2* in FB1*P_crg1_:fuz7DD*, we constructed the plasmid p5HOP2. This plasmid consists in 1 kb fragment of the upstream sequence of *pac2* open reading frame (ORF) followed by the *otef* constitutive promoter, the hygromycin resistance cassette and 1 kb of the *pac2* ORF integrated in a pGEM-T-EASY vector. For this purpose 1 kb fragment of the upstream sequence of *pac2* was amplified with the primers Umpac2-5UTR-1 and Umpac2-5UTR-2, using FB1 genomic DNA; the *otef* constitutive promoter followed by 1kb of *pac2* ORF was amplified with the primers Umotefpac2 and Umpac2-+1kb, using the plasmid p123-*pac2* as template. Both flanks where then digested with *SfiI* restriction enzyme and ligated with the hygromycin resistance cassette. This construction was ligated to a pGEM-T-EASY vector. FB1*P_crg1_:fuz7DDpac^con^* was generated by transformation of the wild-type FB1*P_crg1_:fuz7DD* with the mentioned construct.

Single homologous integration of the linear plasmids or PCR products transformed was verified by PCR and Southern blot.

In the expression analysis, cells grown on liquid culture were recovered by centrifugation, washed with cold water, and total RNA was isolated with QIAGEN (Valencia, CA) RNeasy mini kit. For charcoal grown cells, biomass was recovered and transferred to liquid nitrogen pre-chilled mortars. Total RNA was then extracted from the crushed powder with trizol reagent (Invitrogen) and with the QIAGEN RNeasy mini kit. Isolated RNA was separated by formaldehyde denaturing agarose gel electrophoresis, and transferred overnight by capillary action to nylon membranes. Probes were obtained by PCR with the oligonucleotides indicated in Table S6. Radioactive labelling of PCR generated probes was carried out. Radioactive bands were visualized and quantified using a Molecular Dynamics PhosphoImager.

For qRT-PCR first strand cDNA synthesis was performed using the Transcriptor First Strand cDNA Synthesis Kit (Roche) according to the manufacturer's protocol. As a template for the reaction 1 µg of total RNA was used. Samples were incubated at 50°C for 1 hour. Real-time PCR was performed in a ABIPRISM 7000 Sequence Detection System (Applied Biosystems) using the Power SYBR Green PCR Master Mix according to the manufacturer's protocol. Primers used for detection are shown in Table S6.

### Sequence alignment and domain structure

*U. maydis* Tup1 sequence was obtained from MIPS *U. maydis* DataBase (http://mips.gsf.de/genre/proj/ustilago/). *S. cerevisiae* and *C. albicans* Tup1 sequences were obtained from SGD (http://www.yeastgenome.org/) and CGD (http://www.candidagenome.org/) databases, respectively. The rest of the Tup1 sequences were obtained from the NCBI. Multiple sequence alignments were made with ClustalW2. Domain structure analysis was performed using InterProScan Sequence Search tool from the European Bioinformatics Institute (http://www.ebi.ac.uk/). Pfam retrieved domains were used. Schematic representation of the retrieved domains was performed maintaining proportions of each domain with respect to the whole protein sequence length.

### Fluorimetric measurement of GFP

Cells were grown on nitrate minimal media containing 1% glucose or 1% maltose to an OD_600_ of 0.6–0.8, then pelleted and resuspendend in sterile water to an OD_600_ of 1.0. Fluorescence from 200 µl of cell suspension transferred to a microtiter plate was measured by using a POLARstar Omega fluorescence reader (BMG LABTECH). GFP fluorescence was measured at a wavelength of 485 nm for excitation and 520 nm for emission. Fluorescence was normalized to OD_600_. At least three independent experiments were performed, each measured in triplicate.

### Microscopy

Cell morphology of WGA-stained cells, conjugation tube and *b*-dependent filament formation were analyzed with a Zeiss Apotome microscope.

For *on planta* quantification of filament and appressoria formation in co-infection experiments with *U. maydis* CFP and YFP labelled strains, leaf samples were stained with calcofluor white (Sigma) to visualize fungal material and then checked for CFP or YFP fluorescence. Quantification of filament formation on charcoal plates was performed by fluorescence analysis of colony samples from co-spotted YFP and CFP strains. Post-penetration stages were visualized by WGA-AF 488 and Propidium Iodide (Sigma) staining of infected leaf samples as previously described [93]. Samples were examined using a Leica fluorescence microscope, equipped with a PlanApo x 100 lens and a Deltavision widefield microscope (Applied Precision, Issaquah, WA) equipped with 20, 40, 63 and 100 x lens. Image processing was carried out using Adobe Photoshop CS2.

### DNA array

SG200 and SG200Δ*tup1* cells were grown on YEPSL until exponential phase, then washed twice with sterile water and cultured on minimal charcoal array plates (12.5% Holliday salts, 2% vitamins, 30 mM L-glutamine, 2% glucose, 4% agar and 2% charcoal, pH 7) during 48 hours at 25°C. 144 cm^2^ plates and a cell density of OD_600_ of0.1/cm^2^ was used. DNA-array analysis was performed using custom-designed Affymetrix chips (UstilagoA). Probe sets for the individual genes can be obtained from http://mips.helmholtz-muenchen.de/genre/proj/ustilago/. Target preparation, hybridization and data analysis was performed as described before [94], with the following alterations: total RNA was extracted as commented in DNA and RNA procedures for charcoal growing cells; 5 µg RNA were used for first strand cDNA synthesis at 50°C with Superscript II (Invitrogen); an adjusted P-value of ≤0.01 for the false discovery rate [95] and a change in expression of ≥2 was used for filtering. Expression values were calculated as mean of two biological replicates. Array data can be accessed at GEO/NCBI database (accession number GSE29591).

### Accession numbers

*U. maydis* sequence data can be found in the GenBank/EMBL data libraries under accession numbers XP_759427 for Tup1, XP_762643.1 for Pac2,, XP_756724 for bE1, XP_756725 for bW1, XP_758529 for Mfa1, XP_760967 for Acf1, XP_762479 for Egl1, XP_762172 for Rop1, XP_762530 for Hap2, XP_758660 for Crk1, XP_758860 for Prf1, XP_757661 for Fuz7, XP_760954 for um04807, XP_758669 for um11413, XP_756174 for um00027, XP_759558 for um03411 and XP_758874 for um02727. Other sequences used in this study have the following accession numbers: *S. cerevisiae* Tup1p, NP_010007; *C. albicans* Tup1, AAB63195; *C. neoformans* Tup1, XP_570974; *P. marneffei* TupA, AAL99251; *N. crassa* Rco-1, AAB37245; *A. nidulans* TupA ACD46267; *S. pombe* Tup11, NP_592873; *S. pombe* Tup12, NP_592910.

## Supporting Information

Figure S1 **Sequence alignment of Tup1 proteins from different organisms.** Different conserved domains are indicated. The *U. maydis* Tup1, *S. cerevisiae* Tup1p, *C. albicans* Tup1, *C. neoformans* Tup1p and *P. marneffei* TupA sequences were aligned using ClustalW2. Accession numbers can be found in Methods.
(TIF)

Figure S2 **Growth and morphology of** ***tup1*** **mutants.** In the first row, colony morphology of wild-type and Δ*tup1* strains grown on YPD plates during 24 hours at 28°C are shown (scale bars represent 1 mm). A magnification of each colony is shown (x2). The second and third rows show differential interference contrast (DIC) and fluorescence images of FITC-labeled wheat germ agglutinin (WGA) cells of each strain during exponential phase growth on rich liquid media (scale bar  = 20 µm).
(TIF)

Figure S3 **Disease symptoms caused by wild-type and** ***tup1*** **mutant strains 21 dpi.** Strains and total numbers of infected plants (n) are indicated within the color legend. Seven day old maize seedlings were infected. Symptoms were scored 21 dpi. Tumor categories correspond to: large tumors (>5 mm), medium tumors (1–5 mm) and small tumors (<1 mm). Mean values of three independent experiments and the standard deviation are shown. Asterisk (*) represents statistically significant differences in regard to the wild-type strain.
(TIF)

Figure S4 **Infection rates of FBD11** ***tup1*** **mutants and crosses between Δ** ***tup1*** **and wild-type strains.** (A) Disease symptoms of plants infected with the indicated strains (color legend). The total number of plants infected with each strain (n) is indicated above each column. Tumor categories correspond to: large tumors (>5 mm), medium tumors (1–5 mm) and small tumors (<1 mm). Mean values of three independent experiments are shown. (B) Representative images of the infections for wild-type and *tup1* mutant strains. Scale bars  = 1 cm. (C) Disease symptoms of plants infected with the indicated strains (color legend). The total number of plants infected with each strain (n) is indicated below the color legend. Tumor categories correspond to: large tumors (>5 mm), medium tumors (1–5 mm) and small tumors (<1 mm). Mean values of three independent experiments and the standard deviation are shown. Statistically significant differences are indicated (*). (D) Representative images of maize flowers infected with wild-type or *tup1* mutant strains. Scale bars  = 1 cm. (E) Disease symptoms of plants infected with the indicated strains (color legend). The total number of plants infected with each strain (n) is indicated below the color legend. Tumor categories correspond to: large tumors (>1 cm) and small tumors (<1 cm). Mean values of three independent experiments and the standard deviation are shown. Statistically significant differences are indicated (*).
(TIF)

Figure S5 **Infection rates and quantification of appressoria formation in SG200 wild-type and** ***tup1*** **mutant strains.** (A) Representative images showing the most prevalent tumor category for wild-type and *tup1* mutant infected plants. Scale bars  = 1 cm. (B) Disease symptoms of plants infected with the indicated strains (color legend). The total number of plants infected with each strain (n) is indicated below the color legend. Tumor categories correspond to: large tumors (>5 mm), medium tumors (1–5 mm) and small tumors (<1 mm). Mean values of three independent experiments and the standard deviation are shown. Asterisk (*) represents statistically significant differences in regard to the wild-type strain. (C) Quantification of appressoria formation. A mixture containing equal numbers of SG200CFP and SG200YFPΔ*tup1* cells was inoculated on seven day old maize seedlings. Appressoria formation was visualized by fluorescence microscopy of calcofluor stained leaf samples 16 to 24 hours post-inoculation. CFP or YFP fluorescence was used to determine the strain to which each appressorium belonged. The total number of appressoria counted (n) is indicated at the top (three independent experiments; standard deviation is shown).
(TIF)

Figure S6 **Quantification of** ***b*** **-dependent filament formation in the AB33 background.** (A) Filament formation of AB33 and AB33Δ*tup1* strains in inducing (nitrate) and non-inducing (ammonium) conditions are represented. Color code for each strain (above), media and cell type (below) are indicated. The total number of yeasts/filaments counted for each strain (n) is indicated below the color legend and valid for both media. Quantification was performed 5 hours post-induction. The mean value of three independent experiments and the standard deviation is represented. (B) Length of *b*-dependent filaments produced by wild-type and *tup1* deletion strains (2 independent experiments). Measurement refers only to the filament, not to the original yeast cell.
(TIF)

Figure S7 **Pathogenicity of solopathogenic** ***tup1*** **mutant strains.** Seven days old maize seedlings were infected with the indicated strains (color legend). Disease symptoms caused by wild-type and *tup1* mutant strains were scored 14 dpi. A non-pathogenic FB1 strain was used as control. The total number of infected plants (n) is indicated above each column. Tumor categories correspond to: large tumors (>5 mm), medium tumors (1–5 mm) and small tumors (<1 mm). Mean values of three independent experiments are shown.
(TIF)

Figure S8 **qRT-PCR analysis of** ***bE1*** **expression. **The indicated strains were grown on charcoal-containing media during 48 hours at 25°C. For normalization *act1* gene was used. Expression was calculated relative to the lowest expression value. Shown are the media of three technical replicates. All comparisons are statistically significant, except when comparing CL13 and SG200Δ*tup1*.
(TIF)

Figure S9 **Quantification of conjugation tube formation frequency and length in the FB1** ***P_crg1_:fuz7DD*** **background.** (A) Quantification of the number of cells with conjugation tubes, upon induction of *fuz7DD* allele, in each strain. Expression of the *fuz7DD* allele was induced by a shift from glucose to arabinose containing CM media. Quantification was performed 5 hours post-induction. The total number of cells counted (n) is given above each column. Three independent experiments were performed and the standard deviation is shown. (B) Length of the conjugation hyphae developed by wild-type and *tup1* mutant strains in a FB1*P_crg1_:fuz7DD* background. Total number of hypha counted (n) (top) for each strain (bottom) are indicated (2 independent experiments). Measurement refers only to the filament, not to the original yeast cell.
(TIF)

Figure S10 **GFP expression level driven by the** ***prf1*** **promoter UAS sequence.** HA232 strains were grown in inducing (minimal media with glucose) or repressing (minimal media with maltose) conditions and GFP fluorescence was measured using a POLARstar Omega fluorometer (BMG LABTECH). Mean values of GFP fluorescence relative to OD_600_ from three independent experiments and the standard deviation are shown.
(TIF)

Table S1 **Identity and similarity between** ***U. maydis*** **Tup1 and Tup1 proteins from other organisms.**
(DOC)

Table S2 **Pfam retrieved domain position of Tup1 proteins.**
(DOC)

Table S3 **Altered gene expression by deletion of** ***tup1*** **gene in the SG200 strain.**
(XLS)

Table S4 **Enrichment analysis for GO-categories of Tup1-regulated genes.**
(XLS)

Table S5 ***U. maydis*** **strains used in this study.**
(DOC)

Table S6 **Primers used in this study.**
(DOC)
