# Supplementary figures and images for: The General Transcriptional Repressor Tup1 Is Required for Dimorphism and Virulence in a Fungal Plant Pathogen
Source: PLoS Pathog. 2011 Sep 1;7(9):e1002235. doi: 10.1371/journal.ppat.1002235 (PMC3164652; doi:10.1371/journal.ppat.1002235)

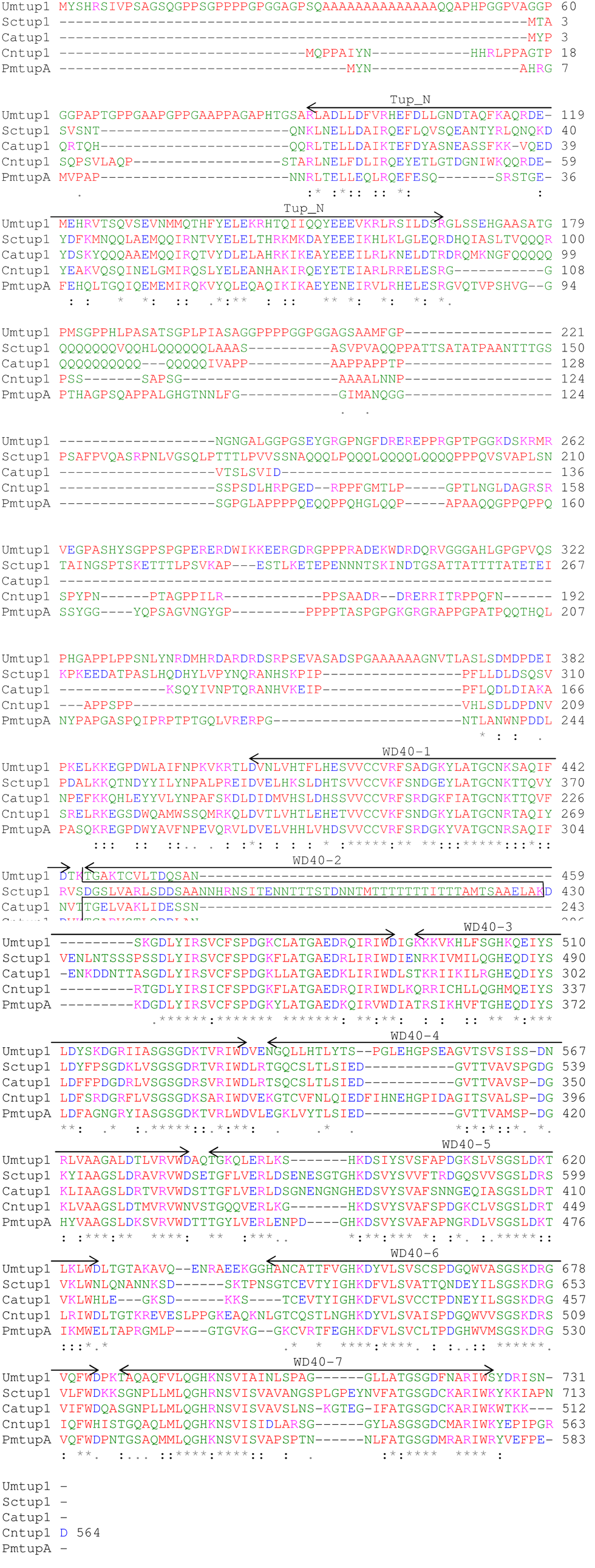

Supplement: Figure S1 — Sequence alignment of Tup1 proteins from different organisms. Different conserved domains are indicated. The U. maydis Tup1, S. cerevisiae Tup1p, C. albicans Tup1, C. neoformans Tup1p and P. marneffei TupA sequences were aligned using ClustalW2. Accession numbers can be found in Methods. (TIF) [file ppat.1002235.s001.tif]

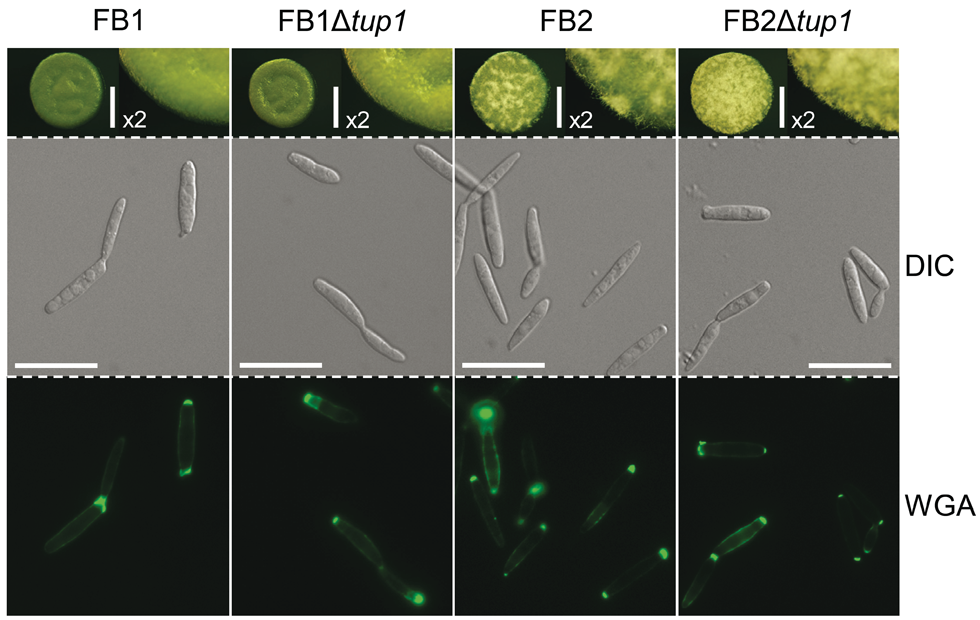

Supplement: Figure S2 — Growth and morphology of tup1 mutants. In the first row, colony morphology of wild-type and Δtup1 strains grown on YPD plates during 24 hours at 28°C are shown (scale bars represent 1 mm). A magnification of each colony is shown (x2). The second and third rows show differential interference contrast (DIC) and fluorescence images of FITC-labeled wheat germ agglutinin (WGA) cells of each strain during exponential phase growth on rich liquid media (scale bar = 20 µm). (TIF) [file ppat.1002235.s002.tif]

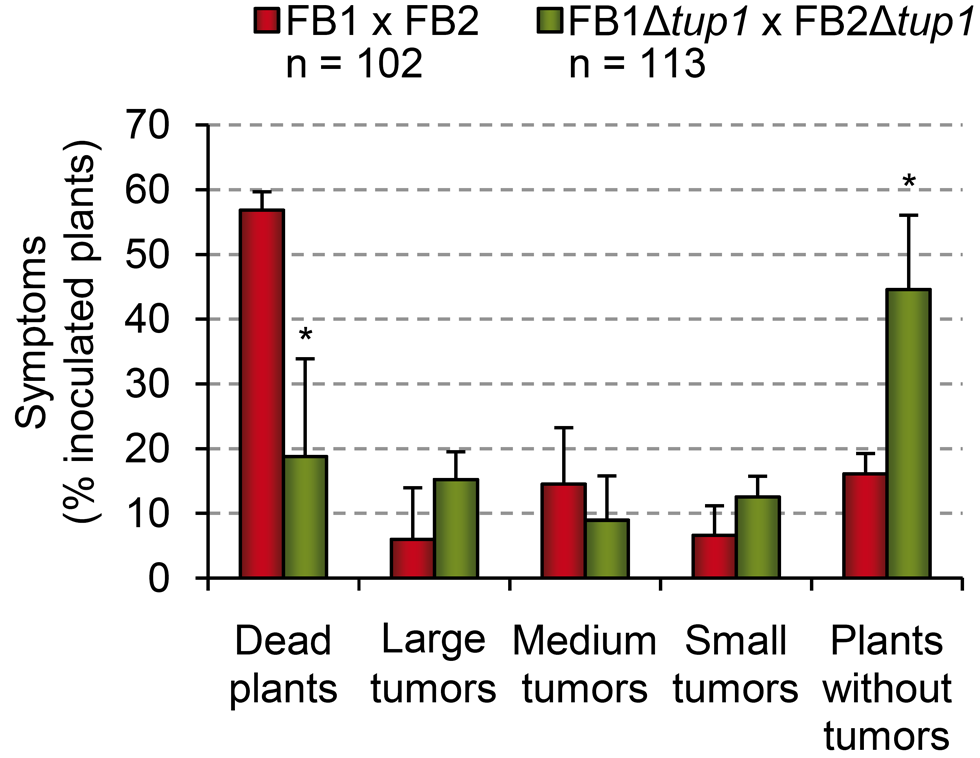

Supplement: Figure S3 — Disease symptoms caused by wild-type and tup1 mutant strains 21 dpi. Strains and total numbers of infected plants (n) are indicated within the color legend. Seven day old maize seedlings were infected. Symptoms were scored 21 dpi. Tumor categories correspond to: large tumors (>5 mm), medium tumors (1–5 mm) and small tumors (<1 mm). Mean values of three independent experiments and the standard deviation are shown. Asterisk (*) represents statistically significant differences in regard to the wild-type strain. (TIF) [file ppat.1002235.s003.tif]

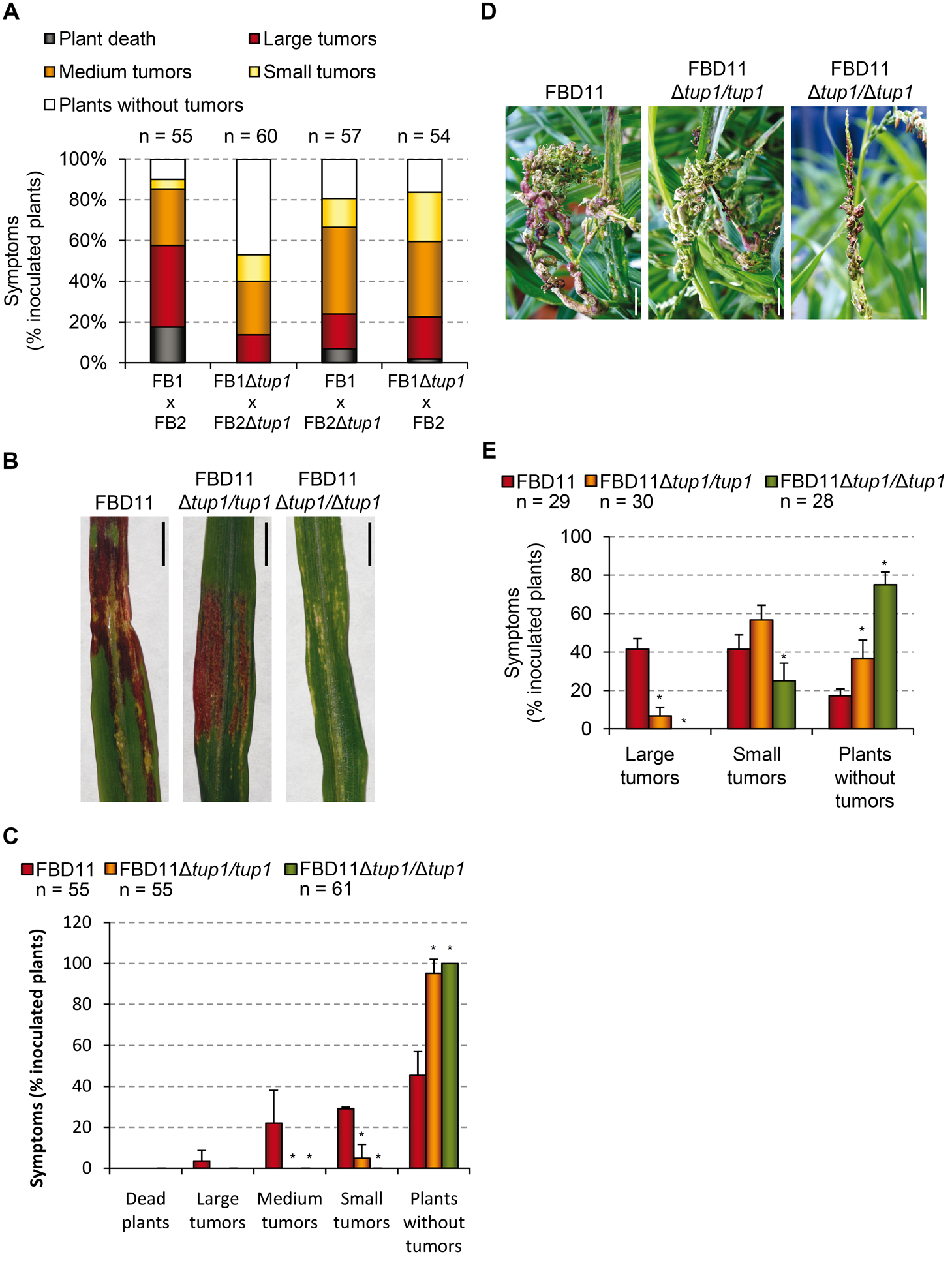

Supplement: Figure S4 — Infection rates of FBD11 tup1 mutants and crosses between Δ tup1 and wild-type strains. (A) Disease symptoms of plants infected with the indicated strains (color legend). The total number of plants infected with each strain (n) is indicated above each column. Tumor categories correspond to: large tumors (>5 mm), medium tumors (1–5 mm) and small tumors (<1 mm). Mean values of three independent experiments are shown. (B) Representative images of the infections for wild-type and tup1 mutant strains. Scale bars = 1 cm. (C) Disease symptoms of plants infected with the indicated strains (color legend). The total number of plants infected with each strain (n) is indicated below the color legend. Tumor categories correspond to: large tumors (>5 mm), medium tumors (1–5 mm) and small tumors (<1 mm). Mean values of three independent experiments and the standard deviation are shown. Statistically significant differences are indicated (*). (D) Representative images of maize flowers infected with wild-type or tup1 mutant strains. Scale bars = 1 cm. (E) Disease symptoms of plants infected with the indicated strains (color legend). The total number of plants infected with each strain (n) is indicated below the color legend. Tumor categories correspond to: large tumors (>1 cm) and small tumors (<1 cm). Mean values of three independent experiments and the standard deviation are shown. Statistically significant differences are indicated (*). (TIF) [file ppat.1002235.s004.tif]

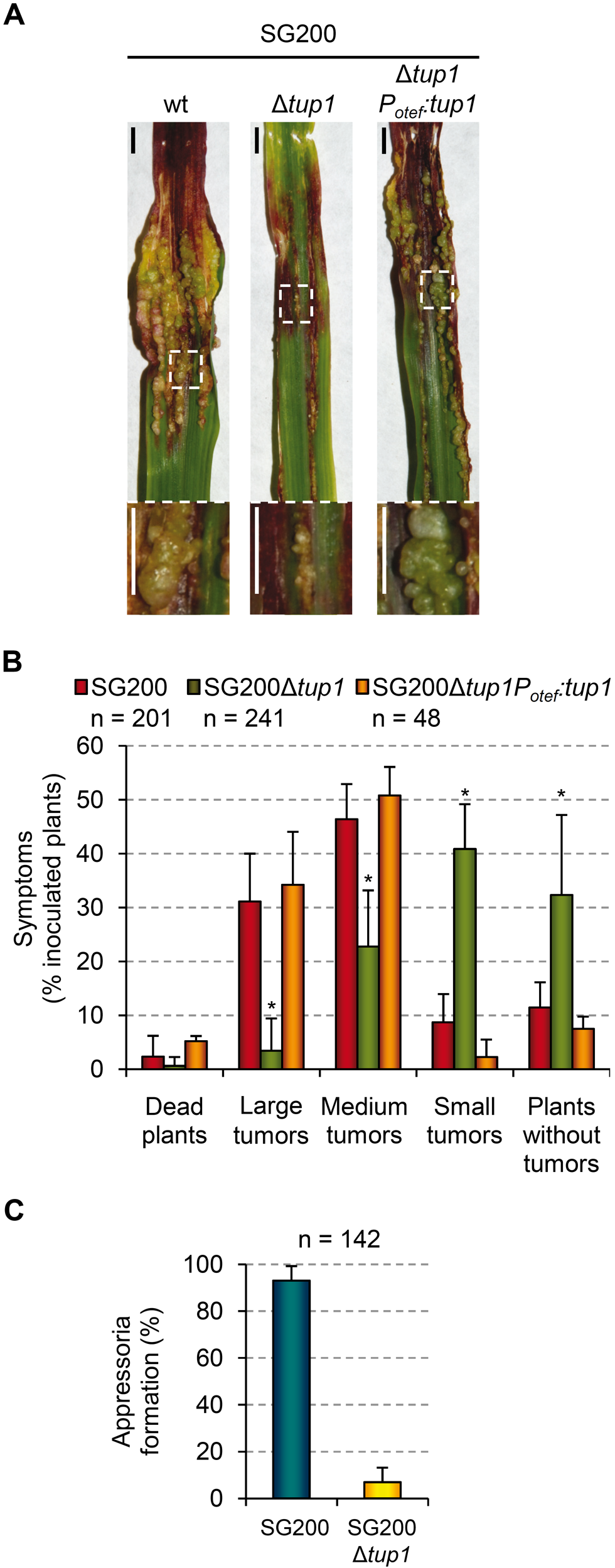

Supplement: Figure S5 — Infection rates and quantification of appressoria formation in SG200 wild-type and tup1 mutant strains. (A) Representative images showing the most prevalent tumor category for wild-type and tup1 mutant infected plants. Scale bars = 1 cm. (B) Disease symptoms of plants infected with the indicated strains (color legend). The total number of plants infected with each strain (n) is indicated below the color legend. Tumor categories correspond to: large tumors (>5 mm), medium tumors (1–5 mm) and small tumors (<1 mm). Mean values of three independent experiments and the standard deviation are shown. Asterisk (*) represents statistically significant differences in regard to the wild-type strain. (C) Quantification of appressoria formation. A mixture containing equal numbers of SG200CFP and SG200YFPΔtup1 cells was inoculated on seven day old maize seedlings. Appressoria formation was visualized by fluorescence microscopy of calcofluor stained leaf samples 16 to 24 hours post-inoculation. CFP or YFP fluorescence was used to determine the strain to which each appressorium belonged. The total number of appressoria counted (n) is indicated at the top (three independent experiments; standard deviation is shown). (TIF) [file ppat.1002235.s005.tif]

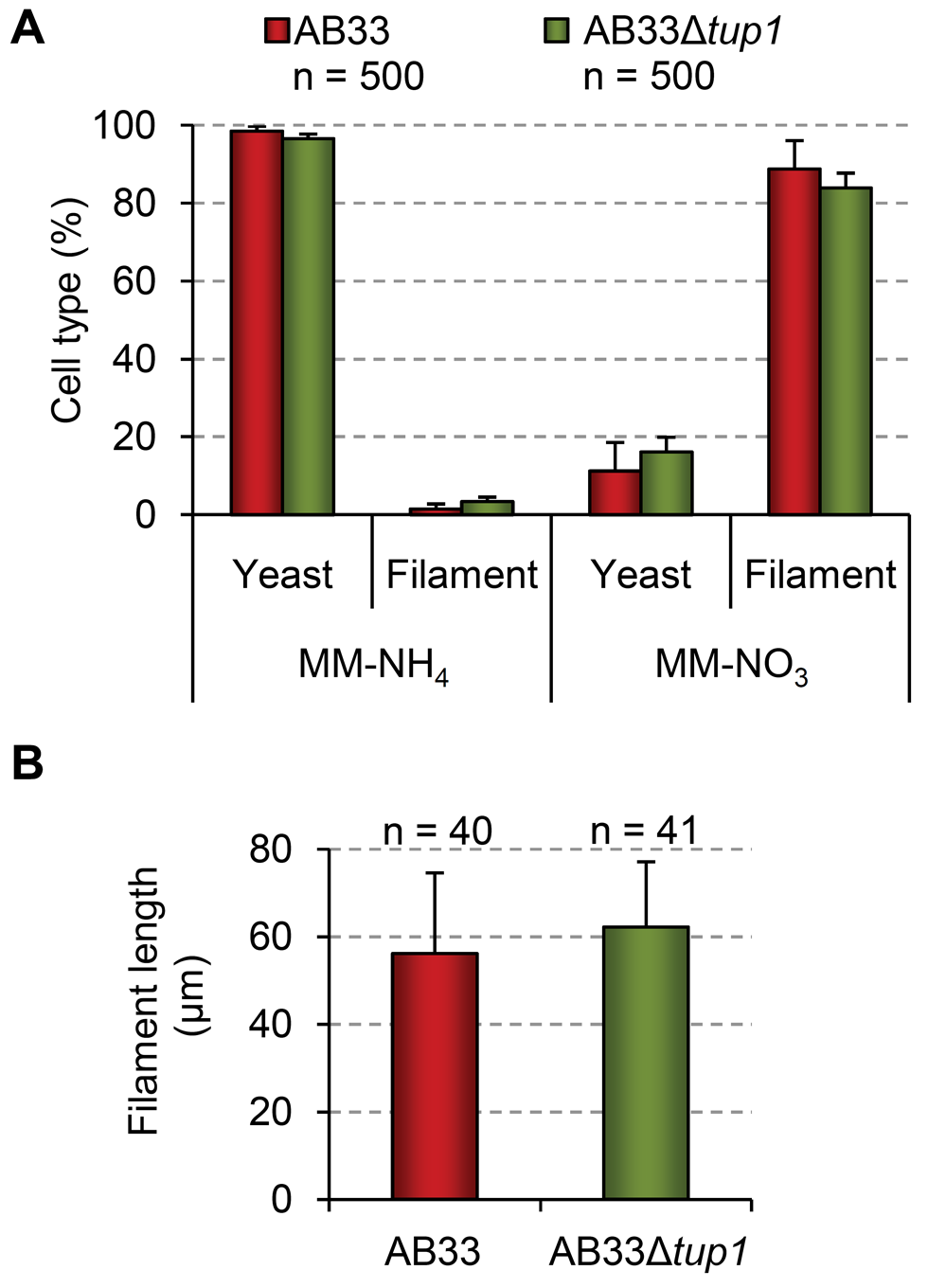

Supplement: Figure S6 — Quantification of b -dependent filament formation in the AB33 background. (A) Filament formation of AB33 and AB33Δtup1 strains in inducing (nitrate) and non-inducing (ammonium) conditions are represented. Color code for each strain (above), media and cell type (below) are indicated. The total number of yeasts/filaments counted for each strain (n) is indicated below the color legend and valid for both media. Quantification was performed 5 hours post-induction. The mean value of three independent experiments and the standard deviation is represented. (B) Length of b-dependent filaments produced by wild-type and tup1 deletion strains (2 independent experiments). Measurement refers only to the filament, not to the original yeast cell. (TIF) [file ppat.1002235.s006.tif]

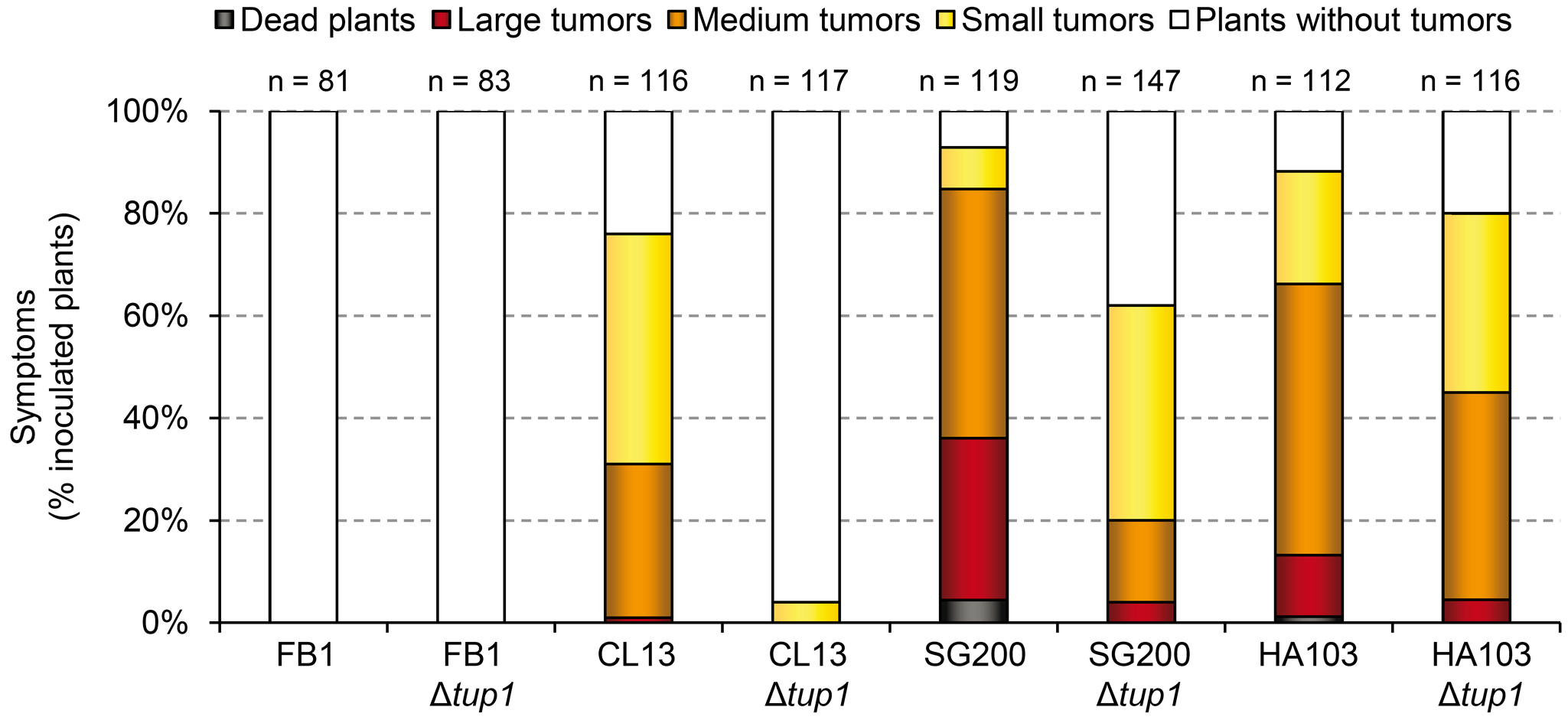

Supplement: Figure S7 — Pathogenicity of solopathogenic tup1 mutant strains. Seven days old maize seedlings were infected with the indicated strains (color legend). Disease symptoms caused by wild-type and tup1 mutant strains were scored 14 dpi. A non-pathogenic FB1 strain was used as control. The total number of infected plants (n) is indicated above each column. Tumor categories correspond to: large tumors (>5 mm), medium tumors (1–5 mm) and small tumors (<1 mm). Mean values of three independent experiments are shown. (TIF) [file ppat.1002235.s007.tif]

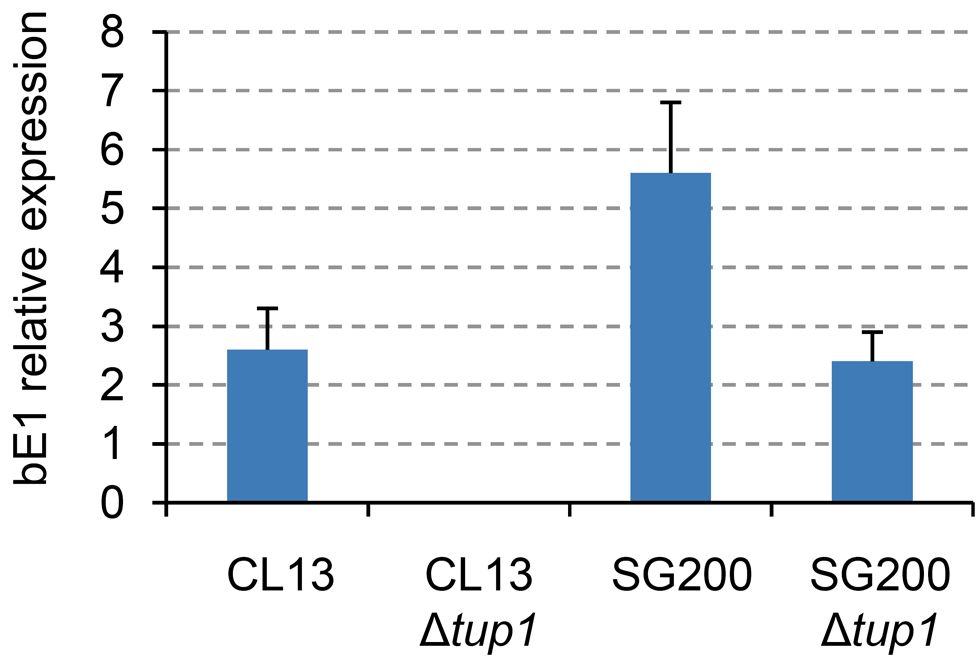

Supplement: Figure S8 — qRT-PCR analysis of bE1 expression. The indicated strains were grown on charcoal-containing media during 48 hours at 25°C. For normalization act1 gene was used. Expression was calculated relative to the lowest expression value. Shown are the media of three technical replicates. All comparisons are statistically significant, except when comparing CL13 and SG200Δtup1. (TIF) [file ppat.1002235.s008.tif]

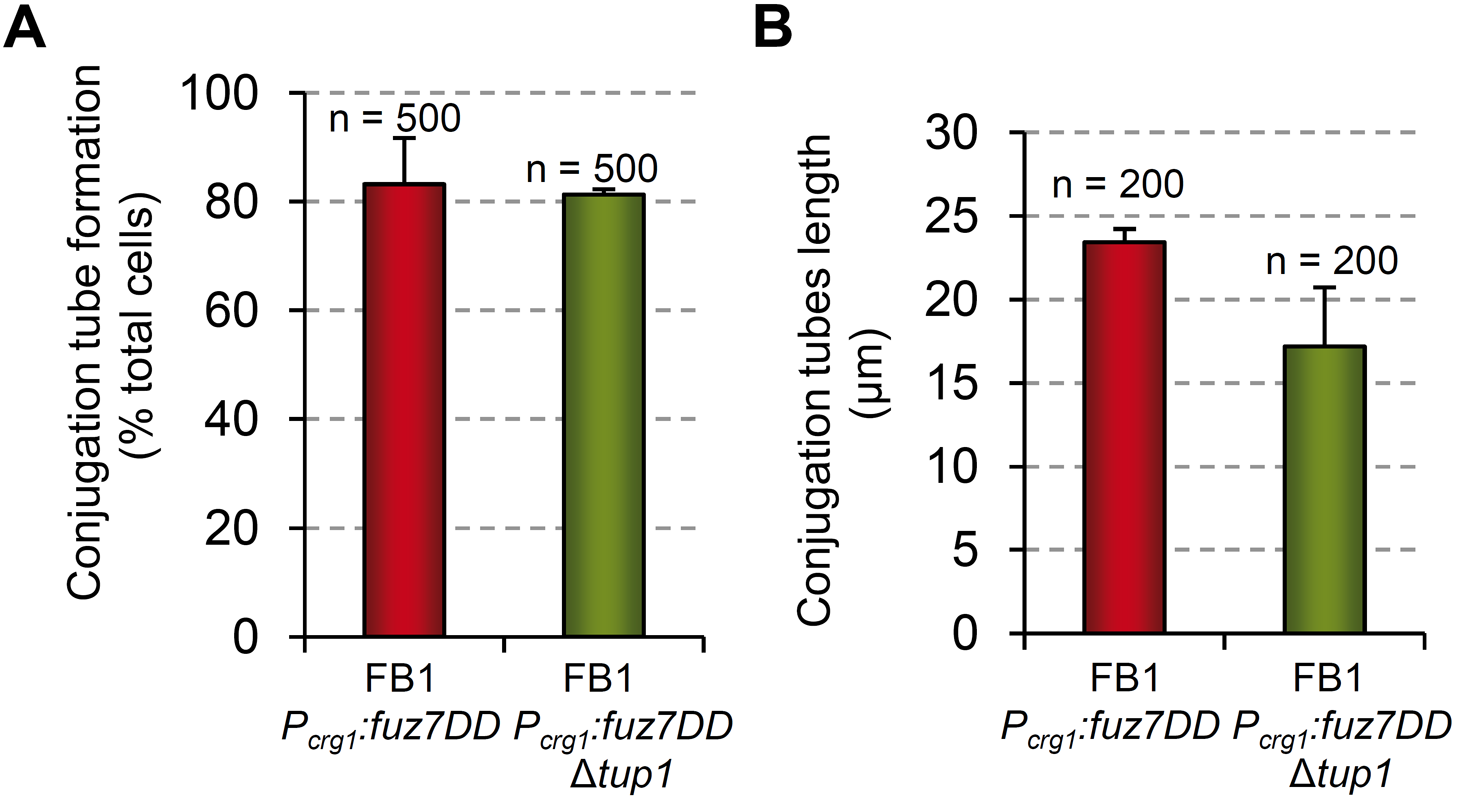

Supplement: Figure S9 — Quantification of conjugation tube formation frequency and length in the FB1 Pcrg1:fuz7DD background. (A) Quantification of the number of cells with conjugation tubes, upon induction of fuz7DD allele, in each strain. Expression of the fuz7DD allele was induced by a shift from glucose to arabinose containing CM media. Quantification was performed 5 hours post-induction. The total number of cells counted (n) is given above each column. Three independent experiments were performed and the standard deviation is shown. (B) Length of the conjugation hyphae developed by wild-type and tup1 mutant strains in a FB1Pcrg1:fuz7DD background. Total number of hypha counted (n) (top) for each strain (bottom) are indicated (2 independent experiments). Measurement refers only to the filament, not to the original yeast cell. (TIF) [file ppat.1002235.s009.tif]

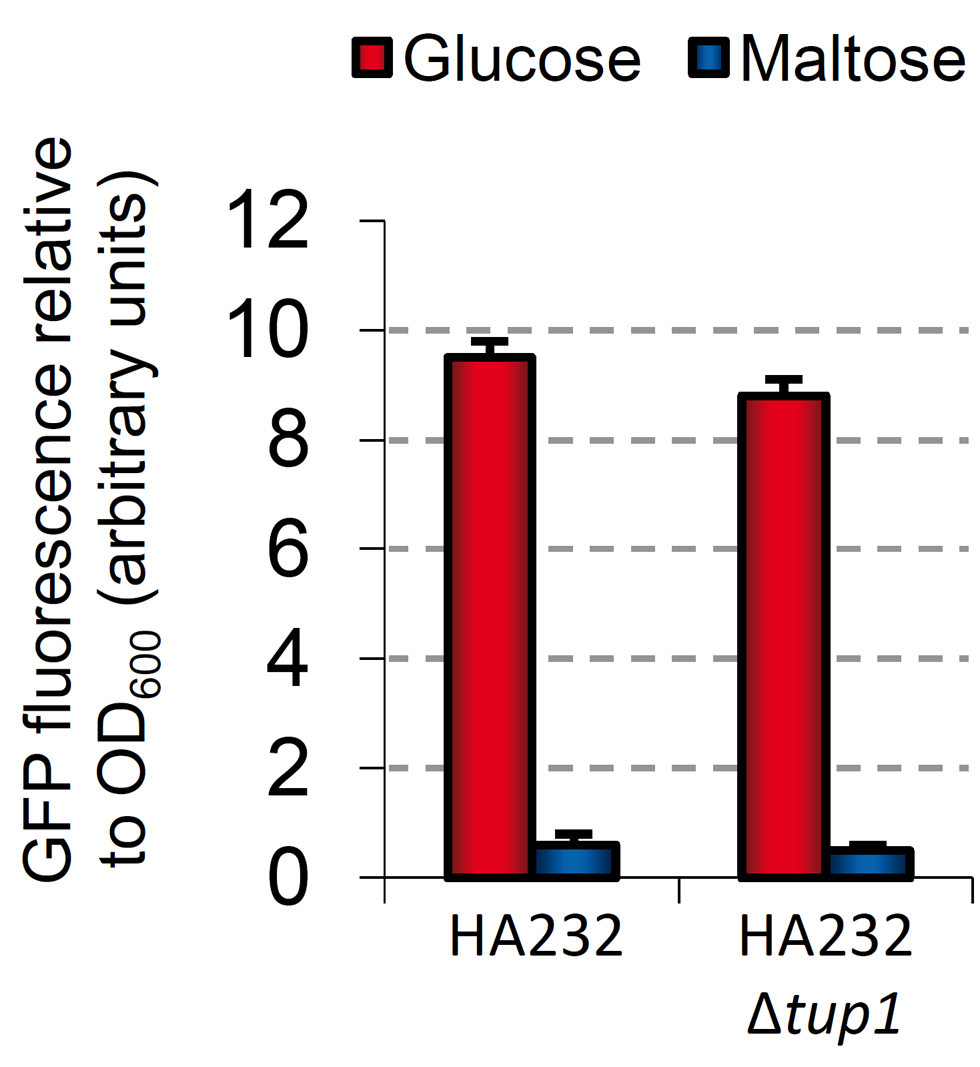

Supplement: Figure S10 — GFP expression level driven by the prf1 promoter UAS sequence. HA232 strains were grown in inducing (minimal media with glucose) or repressing (minimal media with maltose) conditions and GFP fluorescence was measured using a POLARstar Omega fluorometer (BMG LABTECH). Mean values of GFP fluorescence relative to OD600 from three independent experiments and the standard deviation are shown. (TIF) [file ppat.1002235.s010.tif]
