# Supplementary material for: The General Transcriptional Repressor Tup1 Is Required for Dimorphism and Virulence in a Fungal Plant Pathogen
Source: PLoS Pathog. 2011 Sep 1;7(9):e1002235. doi: 10.1371/journal.ppat.1002235 (PMC3164652; doi:10.1371/journal.ppat.1002235)
Supplement: Table S1 — Identity and similarity between U. maydis Tup1 and Tup1 proteins from other organisms. (DOC) [file ppat.1002235.s011.doc]

| **Organism** | **Identity** | **Similarity** | **E value** |
| --- | --- | --- | --- |
| *S. cerevisiae* | 149/298 (50%) | 198/298 (66%) | 9.4e-81 |
| *C. albicans* | 180/374 (49%) | 250/374 (67%) | 2e-100 |
| *C. neoformans* | 238/417 (58%) | 301/417 (73%) | 5e-138 |
| *P. marneffei* | 216/357 (61%) | 267/357 (75%) | 6e-128 |
| *N. crassa* | 219/356 (62%) | 267/356 (75%) | 1e-128 |
| *A. nidulans* | 216/365 (60%) | 266/365 (73%) | 4e-123 |
| *S. pombe* Tup11 | 173/341 (51%) | 239/341 (71%) | 8e-106 |
| *S. pombe* Tup12 | 179/343 (53%) | 241/343 (71%) | 1e-104 |

**Table S1. Identity and similarity between *U. maydis* Tup1 and Tup1 proteins from other organisms.**

Data obtained from MIPS *Ustilago maydis* database (MUMDB).
