# Supplementary material for: The General Transcriptional Repressor Tup1 Is Required for Dimorphism and Virulence in a Fungal Plant Pathogen
Source: PLoS Pathog. 2011 Sep 1;7(9):e1002235. doi: 10.1371/journal.ppat.1002235 (PMC3164652; doi:10.1371/journal.ppat.1002235)
Supplement: Table S2 — Pfam retrieved domain position of Tup1 proteins. (DOC) [file ppat.1002235.s012.doc]

**Table S2: Pfam retrieved domain position of Tup1 proteins.**

| **Organism** | **Domain*** | **Position (aminocids)** |
| --- | --- | --- |
| *U. maydis* | Tup1_N | 91-166 |
|  | WD1 | 396-443 |
|  | WD2 | 446-491 |
|  | WD3 | 494-533 |
|  | WD4 | 536-583 |
|  | WD5 | 586-625 |
|  | WD6 | 644-683 |
|  | WD7 | 686-725 |
| *S. cerevisiae* | Tup1_N | 11-90 |
|  | WD1 | 334-375 |
|  | WD2 | 430-471 |
|  | WD3 | 474-513 |
|  | WD4 | 516-555 |
|  | WD5 | 558-604 |
|  | WD6 | 619-658 |
|  | WD7 | 661-706 |
| *C. albicans* | Tup1_N | 11-89 |
|  | WD1 | 190-227 |
|  | WD2 | 230-283 |
|  | WD3 | 286-325 |
|  | WD4 | 328-366 |
|  | WD5 | 369-415 |
|  | WD6 | 423-462 |
|  | WD7 | 465-508 |
| *C. neoformans* | Tup1_N | 31-108 |
|  | WD1 | 233-270 |
|  | WD2 | 273-318 |
|  | WD3 | 321-360 |
|  | WD4 | 363-412 |
|  | WD5 | 415-454 |
|  | WD6 | 475-514 |
|  | WD7 | 517-556 |
| *P. marneffei* | Tup1_N | 15-86 |
|  | WD1 | 268-305 |
|  | WD2 | 308-353 |
|  | WD3 | 356-395 |
|  | WD4 | 398-436 |
|  | WD5 | 439-481 |
|  | WD6 | 496-535 |
|  | WD7 | 538-577 |

* Tup1 protein sequences were individually pasted into the IterProScan Sequence Search tool at the European Bioinformatics Institute. Pfam retrieved domains were used.
