# Supplementary material for: The General Transcriptional Repressor Tup1 Is Required for Dimorphism and Virulence in a Fungal Plant Pathogen
Source: PLoS Pathog. 2011 Sep 1;7(9):e1002235. doi: 10.1371/journal.ppat.1002235 (PMC3164652; doi:10.1371/journal.ppat.1002235)
Supplement: Table S5 — U. maydis strains used in this study. (DOC) [file ppat.1002235.s015.doc]

Table S5: *U. maydis* strains used in this study.

| **Strain** | **Relevant Genotype** | **Reference** |
| --- | --- | --- |
| FB1 | *a1 b1* | Banuett and Herskowitz (1989) |
| FB2 | *a2 b2* | Banuett and Herskowitz (1989) |
| CL13 | *a1 bW2 bE1* | Bölker *et al* (1995) |
| SG200 | *a1 mfa2 bW2 bE1* | Bölker *et al* (1995) |
| HA103 | *a1* *P_hsp70_:bW2* *P_otef_:bE1* | Hartmann *et al* (1996) |
| AB33 | *a2 P_nar_:bW2 P_nar_:bE1* | Brachmann *et al* (2001) |
| SG200CFP | *a1 mfa2 bW2 bE1 P_OMA_:CFP* | Flor-Parra *et al* (2006) |
| SG200YFP | *a1 mfa2 bW2 bE1 P_OMA_:YFP* | Flor-Parra *et al* (2006) |
| FB1*P_crg1_:fuz7DD* | *a1 b1 P_crg1_:fuz7DD* | Müller *et al* (2003) |
| HA232 | *a2 b2* UAS:GFP (a2b2 B-UAS3/cbx) | Hartmann *et al* (1999) |
| SG200*prf1^con^* | *a1 mfa2 bW2 bE1 prf1^tef^* | Garrido *et al* (2004) |
| FB1Δ*tup1* | *a1 b1* Δ*tup1* | This work |
| FB2Δ*tup1* | *a2 b2* Δ*tup1* | This work |
| CL13Δ*tup1* | *a1 bW2 bE1* Δ*tup1* | This work |
| SG200Δ*tup1* | *a1 mfa2 bW2 bE1* Δ*tup1* | This work |
| SG200Δ*tup1P_otef_:tup1* | *a1 mfa2 bW2 bE1* Δ*tup1 P_otef_:tup1* | This work |
| HA103Δ*tup1* | *a1 P_hsp70_:bW2 P_otef_:bE1* Δ*tup1* | This work |
| SG200YFPΔ*tup1* | *a1 mfa2 bW2 bE1 P_OMA_:YFP* Δ*tup1* | This work |
| AB33Δ*tup1* | *a2 P_nar_:bW2 P_nar_:bE1* Δ*tup1* | This work |
| FB1*P_crg1_:fuz7DD*Δ*tup1* | *a1 b1 P_crg1_:fuz7DD* Δ*tup1* | This work |
| FB1*P_crg1_:fuz7DD*Δ*pac2* | *a1 b1 P_crg1_:fuz7DD* Δ*pac2* | This work |
| FB1*P_crg1_:fuz7DD*Δ*tup1*Δ*pac2* | *a1 b1 P_crg1_:fuz7DD* Δ*tup1*Δ*pac2* | This work |
| FB1*P_crg1_:fuz7DDpac2^con^* | *a1 b1 P_crg1_:fuz7DD P_otef_:pac2* | This work |
| SG200Δ*pac2* | *a1 mfa2 bW2 bE1* Δ*pac2* | This work |
| SG200*pac2^con^* | *a1 mfa2 bW2 bE1 P_otef_:pac2* | This work |
| SG200Δ*tup1*Δ*pac2* | *a1 mfa2 bW2 bE1* Δ*tup1*Δ*pac2* | This work |
| SG200Δ*04807* | *a1 mfa2 bW2 bE1* Δ*04807* | This work |
| HA232Δ*tup1* | *a2 b2* UAS:GFP Δ*tup1* | This work |
| SG200*prf1*^con^Δ*tup1* | *a1 mfa2 bW2 bE1 prf1^con^* Δ*tup1* | This work |
