# Supplementary material for: The General Transcriptional Repressor Tup1 Is Required for Dimorphism and Virulence in a Fungal Plant Pathogen
Source: PLoS Pathog. 2011 Sep 1;7(9):e1002235. doi: 10.1371/journal.ppat.1002235 (PMC3164652; doi:10.1371/journal.ppat.1002235)
Supplement: Table S6 — Primers used in this study. (DOC) [file ppat.1002235.s016.doc]

**Table S6: Primers used in this study.**

| **Primer** | **Sequence (5’-3’)** |
| --- | --- |
| *tup1* deletion | |
| UmTUP1KO5-1 | TCTTCGTTGTCGATGTGTCG |
| UmTUP1KO5-2 | GCAGATGGCACGATAGAACGCACGGCCTGAGTGGCC |
| UmTUP1KO3-1 | GTGGGCCATCTAGGCCCAGAATCTGGAGCTACGACC |
| UmTUP1KO3-2 | AGCCTAAAGTCACGGTGGAGG |
| *pac2* deletion | |
| UmPAC2KO5-1 | CTTCCATTTCATCAGCGAGTATTGG |
| UmPAC2KO5-2 | CACGGCCTGAGTGGCCTGTGCCAAAGAACGATCTGCAAATGG |
| UmPAC2KO3-1 | GTGGGCCATCTAGGCCACATTTGGGTCTTCTCGGTTCTCG |
| UmPAC2KO3-2 | ACAGACAGTCACGAGTCTTGATTCG |
| *04807* deletion | |
| Um04807KO5-1 | TTGCACTCTTGTCTCTTGCACC |
| Um04807KO5-2 | CACGGCCTGAGTGGCCTGCACCAATATGAAGTCGAGACG |
| Um04807KO3-1 | GTGGGCCATCTAGGCCTTGTTCTTCCACCAGCTCTCTATGG |
| Um04807KO3-2 | GTTCGACATTGATGCGTTGATGG |
| *tup1* complementation | |
| Umtup1-Stop | CTTAAGTTACGATCAATCACAGAAAATCG |
| Umtup1-Start | CCATGGATGTATTCTCACCGTTCTATCGTGC |
| *pac2* over-expression (ip locus) | |
| UmPac2ATGSmaXma | TAACCCGGGCTGACGGAATTCGATCCCATGCCAGGTCCTCACAGCAACATCTCG |
| UmPac2StopNotI | TAAGCGGCCGCTCAGATGCGAACGCTGAATCGGTCGA |
| *pac2* constitutive expression (endogenous locus) | |
| Umpac2-5UTR-1 | TTCTTCATAGTCCGCTTCATCCTTGCTGACG |
| Umpac2-5UTR-2 | CACGGCCTGAGTGGCCTGTGCCAAAGAACGATCTGCAAATGGACTGAGAGG |
| Umotefpac2 | GTGGGCCATCTAGGCCCATCAGAGCAGATTGTACTGAGAGTGCACC |
| Umpac2-+1kb | ACTGACCAGCTCATGTCTTGAAGCAGACTCG |
| Northern probes | |
| Prf1-1 | TCCTCGCAATGCCTACATCATCTTTCG |
| Prf1-2 | AATCGTCTTGCGACCTTTCCACCTTGG |
| Mfa1-1 | TTGAGACAAGCGAAGTCCATCTTCTGC |
| Mfa1-2 | TGTTGATATCACGTGTCAAGGCATTCG |
| bE1-1 | AGAAACCCTCGTTAGGTTGACCAACG |
| bE1-2 | TGTCGAACACATGCTGAGTTCTGG |
| bW2-1 | AGCAAGTCACTATCTGGTTTCAGAACC |
| bW2-2 | ATCTGTCCAAAGCCTTGCTGTTGC |
| Egl1-1 | ATGGCCTTCAAGCTCAACATCG |
| Egl1-2 | GATGCATCACCGTTAGAAGAACC |
| Rop1-1 | ATGATCGTGCTGAAACAGCAGAGC |
| Rop1-2 | AGTTGGCGATTGTGGTGTAGC |
| Crk1-1 | ATGACGCCTTTCTCATGCCTACC |
| Crk1-2 | TTGACTCTGACGATGGGAGAACC |
| Hap2-1 | ACATCTGTACGAGCCTATGAGC |
| Hap2-2 | TTGACATCAACGGCATCGTGATCG |
| Pac2-1 | ATCGAGGTCACATCAAGACC |
| Pac2-2 | TCTTGGGTCAGGAGACAAGC |
| 00027-1 | TTGGTTGACCATGCGTCACTCG |
| 00027-2 | TCGTTGGCAGAGTAGAGGATCG |
| 02727-1 | TTCAAGTCATCCTCGTTCTCG |
| 02727-2 | GTCACTGTATTGCCAAAAGG |
| 02758-1 | AGTTCTACGATGACAGCATCC |
| 02758-2 | AGCACACTCGGTTTCATAAACC |
| 03411-1 | TCAACGAGAACGGTTCATTCC |
| 03411-2 | AACGCAGTTCTTCTGGTTGAGG |
| 11063.2-1 | TACTGCCTCATTCAGCTCTACATGC |
| 11063.2-2 | ATCTTGATCGTAGGTGAGCGAAGG |
| 11413-1 | AGTACGAGATTTGCATCTGCAAGC |
| 11413-2 | TACATAGCGGAAGAAACGGTTCG |
| Act1-1 | TCCAAGCGTGGTATTTTGACG |
| Act1-2 | GTACTCCTGCTTCGAGATCC |
| qRT-PCR | |
| RTbE1-1 | GGAGGATCTTCCTGCGTATCAC |
| RTbE1-2 | TGGACTGGCCAACTCTTGCT |
| RTAct1-1 | CTCGGGTGACGGTGTTACG |
| RTAct1-2 | AGCCTCGCTCCGTCAAGAT |
